# Transcriptional responses of wheat and the cereal cyst nematode *Heterodera avenae* during their early contact stage

**DOI:** 10.1038/s41598-017-14047-y

**Published:** 2017-11-03

**Authors:** Changlong Chen, Lei Cui, Yongpan Chen, Hongjun Zhang, Pei Liu, Peipei Wu, Dan Qiu, Jingwei Zou, Dan Yang, Li Yang, Hongwei Liu, Yang Zhou, Hongjie Li

**Affiliations:** 1 The National Key Facility for Crop Gene Resources and Genetic Improvement, Institute of Crop Sciences, Chinese Academy of Agricultural Sciences, Beijing, 100081 China; 2 Department of Plant Pathology, China Agricultural University, Beijing, 100193 China

## Abstract

Cereal cyst nematode (*Heterodera avenae*) is attracted to and aggregated around wheat roots to initiate infection, but this interaction between wheat and the nematode is not fully understood. The transcriptional responses of both wheat and *H. avenae* were examined during their early contact stage by mRNA sequencing analysis; certain numbers of the differentially expressed genes (DEGs) were validated using quantitative real-time PCR. The immobile host wheat root only had 93 DEGs (27 up-regulated and 66 down-regulated), while the mobile plant parasitic nematode reacted much more actively with 879 DEGs (867 up-regulated and 12 down-regulated). Among them, a number of wheat DEGs (mostly down-regulated) were involved in biotic stress pathways, while several putative effector genes were up-regulated in the nematode DEGs. One putative chitinase-like effector gene of *H. avenae* was able to suppress BAX-triggered programmed cell death in *Nicotiana benthamiana*. Results of these experiments demonstrated that nematode responded more actively than wheat during the contact stage of parasitism. The parasite’s responses mainly involved up-regulation of genes including at least one anti-plant-defence effector gene, whereas the host responses mainly involved down-regulation of certain defence-related genes.

## Introduction

Plant-parasitic nematodes (PPNs) have caused extensive damage to many plant species^1^. Cyst nematodes, such as *Heterodera* spp. and *Globodera* spp. on cereal crops and soybean (*Glycine max* (L.) Merr.), and root-knot nematode (RKN, *Meloidogyne* spp.), are the most widely studied species because of their economic importance^2^. *Heterodera avenae* Wollenweber is globally one of the most important species of the cereal cyst nematodes (CCNs). It occurs in about 80% of the wheat (*Triticum aestivum* L.) growing areas in China^3^. Infestation of CCN has caused substantial yield losses of wheat ranging from 30 to 100%^4,5^. The discovery of the mechanisms underlying plant-nematode interactions will provide clues on the control of this destructive nematode.

Many parasitic nematodes produce larvae, which use sensory cues for locating their hosts. This complex behavior of the nematodes involves different sensory capabilities, for example olfaction and gustation, as well as temperature and humidity sensing. Although preventative measures can be applied during the attraction of the nematodes to plant roots, this initial step in the parasite-host interactions remains poorly understood^6^. PPNs can be attracted to plant roots^7^ and both volatile and non-volatile root extracts have been shown to attract potato cyst nematode (*G. pallida* Stone)^8^. Ethylene and auxin signaling pathways affect the attraction or repulsion of the roots to nematodes^2,9–11^. Increases in ethylene production reduced the attraction to the host by RKN^10^, while high auxin concentrations attracted *Aphelenchoides besseyi* Christie^11^ and RKN^2^. This might also be due to possible cross-talk between auxin and ethylene in plant^12^.

*Meloidogyne* species are attracted to low pH at the levels similar to the low pH environment produced by the growing roots^13^. Carbon dioxide (CO_2_) attracts a number of PPN species^14–18^. Plant-parasitic nematodes are also attracted to certain root volatiles, which are identical to those emitted by insect-damaged plants to attract entomopathogenic nematodes^6,19^. Nevertheless, little is known about the responses of either parasitic nematodes or their hosts during host-attraction/contact process. It was reported that RKN could activate subcellular reorganization and root-hair deformation in *Lotus japonicus* and tomato (*Solanum lycorpersicum* L.) roots via a signal that could be transmitted at a distance from the host^20^.

Studies on plant-nematode interactions have taken advantage of high-throughput techniques such as transcriptome sequencing, e.g., RKN^21–27^, cyst nematodes^28–34^ and other PPNs^24,35^. The first *de novo* transcriptomic analysis compared the gene expression of pre-parasitic infective juveniles (J2s) to adults in *H. avenae*, which resulted in the identification of some important genes that may be involved in either plant parasitism or nematode metabolism^30^. During the interaction between CCN and *Aegilops variabilis* Eig., the transcriptome of both CCN and *Ae. variabilis* roots were analyzed at 30 h, 3 d, and 9 d post inoculation^28,32^. Those studies identified 7,408 unigenes and three pathways in *Ae. variabilis* associated with plant stress resistance. They also detected 681 putative genes in the parasitic nematode, which included 56 putative effectors. Comparative transcriptome analysis of susceptible and resistant wheat cultivars was used to study the defence responses of wheat against *H. avenae* during the early infection stages (i.e., 24 h, 3 d and 8 d post infection), which resulted in the identification of 606 resistance genes and diverse defence-related pathways^31^.

To date, no transcriptome analysis has focused on the stage before infection of nematodes to their hosts, the initial contact stage of the host-nematode interaction. Previous studies have reported that *H. filipjevi* and *H. avenae* were attracted to and aggregated around wheat roots^36,37^. This study was conducted to identify transcriptomic responses of both wheat and its parasite *H. avenae* during the initial contact stage.

## Results

### Attraction of *H. avenae* to roots of wheat cultivar Wenmai 19

**Figure 1:**
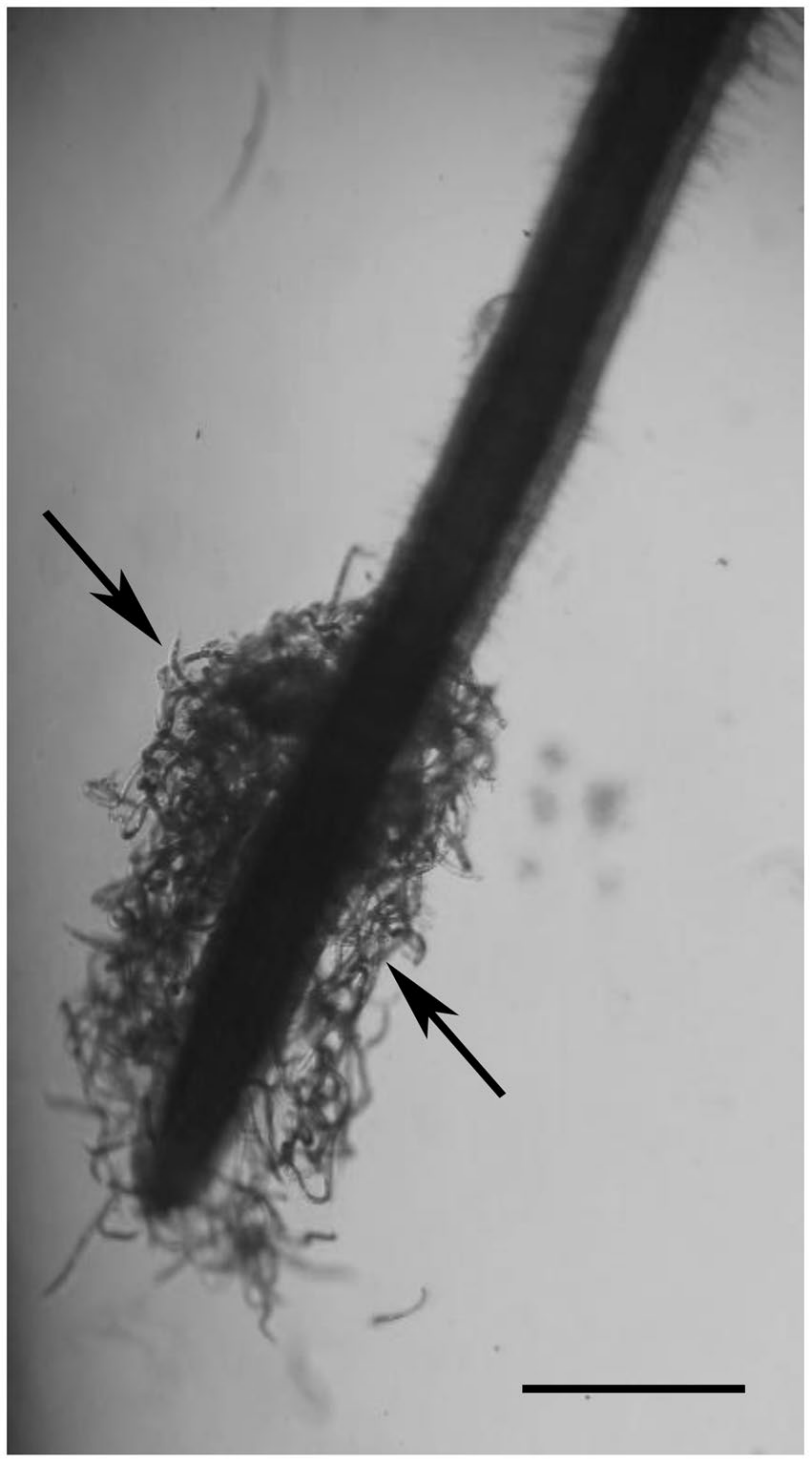
Attraction of *Heterodera avenae* juveniles to the root tips of Wenmai 19 wheat. The figure shows the aggregation of *H. avenae* juveniles (indicated by arrows) around a wheat root tip when nematodes and wheat roots were incubated together in a Pluronic F-127 gel for 3 hours (scale bar = 1 mm).

The CCN-susceptible wheat cultivar Wenmai 19 was used to attract infective J2s of *H. avenae*. The J2 nematodes, which were scattered in the Pluronic F-127 gels, migrated toward wheat roots and gathered in a large quantity around the root tips after 3 h (Fig. 1), except for a few that remained away from the wheat roots. The numbers of J2s gathering around wheat root tips peaked at 3 h, so samples of the wheat roots and the nematodes were collected at this time for transcriptome analysis. Simultaneously, it was confirmed that the *H. avenae* J2s did not penetrate the wheat roots, as no nematodes were observed inside the stained roots (data not shown).

### Transcriptome data from the wheat roots

Six samples of wheat root tips, i.e., three replicates from wheat that had attracted nematodes (wheat treatment) and three from the wheat control, were separately subjected to RNA-sequencing analysis. A total of 52.23 Gb of clean data were obtained from the six root samples altogether, each of which contained ≥8.25 Gb with Quality Scores of Q30 ≥ 90.4% (Supplementary Table S1). A range of 64.0% to 67.3% clean reads of each sample were aligned onto the wheat reference genome and matched to either unique or multiple genomic locations (Table 1). In total, 109,496 unigenes including 9,152 new genes were mined in the wheat transcriptome. According to the databases of Non-redundant protein sequences (Nr), Swiss-Prot, Gene Ontology (GO), Kyoto Encyclopedia of Genes and Genomes (KEGG) and Clusters of Orthologous Groups (COG), 6,780 new genes were annotated. The replicates of wheat roots repeated well with each other (*r* ^2^ = 0.98~1.00) (Supplementary Fig. S1a).

**Table 1:** Summary of read numbers aligned onto the wheat reference genome in the study.

Samples	Total reads	Mapped reads	Uniquely mapped reads	Multiple mapped reads
Control-wheat-R1	57,503,228	38,677,956 (67.3)	32,083,721 (55.8)	6,594,235 (11.5)
Control-wheat-R2	58,077,944	37,182,742 (64.0)	32,331,538 (55.7)	4,851,204 (8.4)
Control-wheat-R3	59,987,522	39,157,955 (65.3)	32,917,113 (54.9)	6,240,842 (10.4)
Treatment-wheat-R1	55,886,834	36,271,171 (64.9)	29,487,722 (52.8)	6,783,449 (12.1)
Treatment-wheat-R2	59,764,690	38,833,790 (65.0)	33,890,672 (56.7)	4,943,118 (8.3)
Treatment-wheat-R3	62,489,824	41,109,281 (65.8)	35,179,867 (56.3)	5,929,414 (9.5)

The number in brackets indicates the percentage of total reads aligned onto the wheat reference genome and/or matched at either unique or multiple genomic locations.

### Wheat genes responding to CCN aggregation

**Figure 2:**
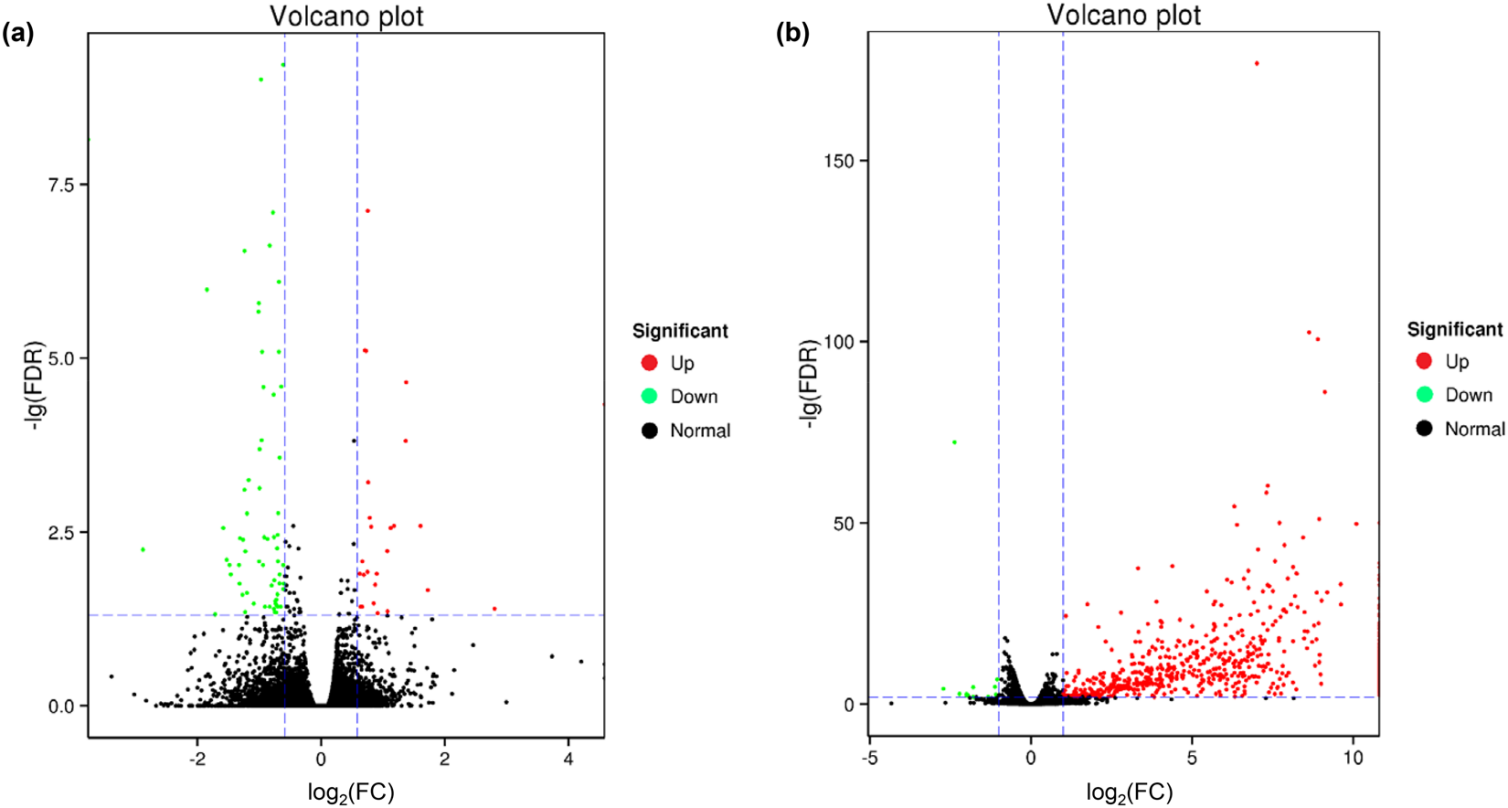
Volcano plots of differentially expressed genes (DEGs) between the nematode-free wheat roots and those exposed to *Heterodera avenae* juveniles (**a**), and wheat root-free *H. avenae* juveniles and those exposed to wheat roots (**b**). Each dot represents one gene with the y-axis showing -lg (FDR) and the x-axis showing log_2_ (FC), respectively. The red, green and normal dots represent the up-regulated DEGs, down-regulated DEGs (FDR < 0.05 and FC ≥ 1.5 for wheat; FDR < 0.01 and FC ≥ 2 for nematode) and not significantly changed genes, respectively. FC: fold change; FDR: false discovery rate.

Comparative analysis of gene expression was performed for the wheat roots that were exposed to the J2 nematodes and the negative control that did not contact with the J2s. Ninety-three unigenes, including 66 down-regulated and 27 up-regulated ones, were differentially expressed with false discovery rate (FDR) < 0.05 and fold change (FC) ≥ 1.5 (Fig. 2, Supplementary Table S2). Twelve differentially expressed genes (DEGs) were validated by quantitative real-time PCR (qPCR) and the expression patterns of eleven DEGs were consistent with those of the mRNA-Seq analysis (Table 2). These results demonstrated that the wheat roots responded to nematode aggregation even though they were not infected by the J2s. Results of the functional annotation indicated that all the DEGs had significant matches in the Nr database, and some of them also had annotation information in the Swiss-Prot, GO, KEGG and COG databases (Table 3).

**Table 2:** Validation of mRNA-Seq data of a selected set of wheat and *Heterodera avenae* genes with quantitative real-time PCR (qPCR) to confirm gene expression changes at the contact stage (treatment versus control).

Gene ID	Annotation	Log_2_FC	
		qPCR	mRNA-seq
**Wheat DEGs**			
gene:TRAES3BF074000020CFD_g	Unnamed protein product [*Triticum aestivum*]	0.01	Inf (up)*
gene:Traes_1AS_F9013A945^a^	Phenylalanine ammonia-lyase [*Aegilops tauschii*]	−0.76*	−1.17*
gene:Traes_2AL_8394449B2	Ubiquinol oxidase 1, mitochondrial (Precursor) [*Nicotiana tabacum*]	−1.19*	−1.27*
gene:Traes_2AS_EE549925C	Root peroxidase [*T. aestivum*]	−0.36	−0.67*
gene:Traes_3DL_EE0699FDC	Secologanin synthase [*Ae. tauschii*]	2.50*	2.80*
gene:Traes_6DS_768787FF4^a^	Auxin-induced protein [*Ae. tauschii*]	−0.83*	−1.31*
gene:Traes_5bs_bcc1b9791^a^	Respiratory burst oxidase homolog protein B [*Oryza sativa* subsp. *japonica*]	0.70*	0.73*
gene:Traes_4bl_eb96605ed^a^	Agmatine coumaroyltransferase-2 [*Hordeum vulgare*]	−0.89*	−0.76*
gene:Traes_4al_dd83f1a44^a^	Xylanase inhibitor [*T. aestivum*]	−0.92*	−0.71*
gene:Traes_2al_1a870ce7b^a^	Probable aldo-keto reductase 3 [*O. sativa* subsp. *japonica*]	−0.56*	−0.61*
gene:Traes_4bs_63dd9d036^a^	Lipoxygenase [*T. aestivum*]	0.21	−0.67*
gene:Traes_1bl_04b591073^a^	NEP1-interacting protein 2 [*Arabidopsis thaliana*]	0.27	1.07*
**Nematode DEGs**			
c73395.graph_c0	Macrophage migration inhibitory protein [*Eriocheir sinensis*]	−0.91	−2.00*
c62312.graph_c0	Programmed cell death protein 2 [*Toxocara canis*]	−2.32*	−1.05*
c73973.graph_c0	Neprilysin-2 [*T. canis*]	1.15*	1.08*
c78521.graph_c0	Fatty acyl-CoA desaturase, putative [*Eimeria tenella*]	2.42*	1.16*
c54125.graph_c0	Sialin [*Ascaris suum*]	1.99*	1.75*
c72543.graph_c0^b^	Pectate lyase [*Heterodera glycines*]	2.17*	1.94*
c76930.graph_c0	Putative salivary protein [*Culicoides sonorensis*]	6.49*	2.09*
c79218.graph_c0	Predicted: transmembrane BAX inhibitor motif-containing protein 4-like [*Amphimedon queenslandica*]	Inf (up)	2.46*
c68622.graph_c0^b^	Chitinase [*H. glycines*]	3.60*	3.32*
c78853.graph_c0^b^	Cathepsin L2 [*Sinonovacula constricta*]	0.77*	1.83*

Inf (up) indicates that the expression of the gene was detected only in the treatment sample, but not the control sample. *P < 0.05. FC, fold change (treatment vs. control). ^a^The DEGs were involved in biotic stress pathways of wheat created using MapMan visualization. ^b^The DEGs were predicted effector genes in *Heterodera avenae* exposed to wheat roots.

**Table 3:** Number of functional annotations of the differentially expressed genes (DEGs) of wheat roots and *Heterodera avenae*, respectively, in the study.

Annotated databases	DEGs number	
	Wheat	*H. avenae*
Nr	93	574
Swiss-Prot	71	410
GO	78	258
KEGG	34	325
COG	29	386
KOG	—	480
Pfam	—	718
Total	93	742

**Figure 3:**
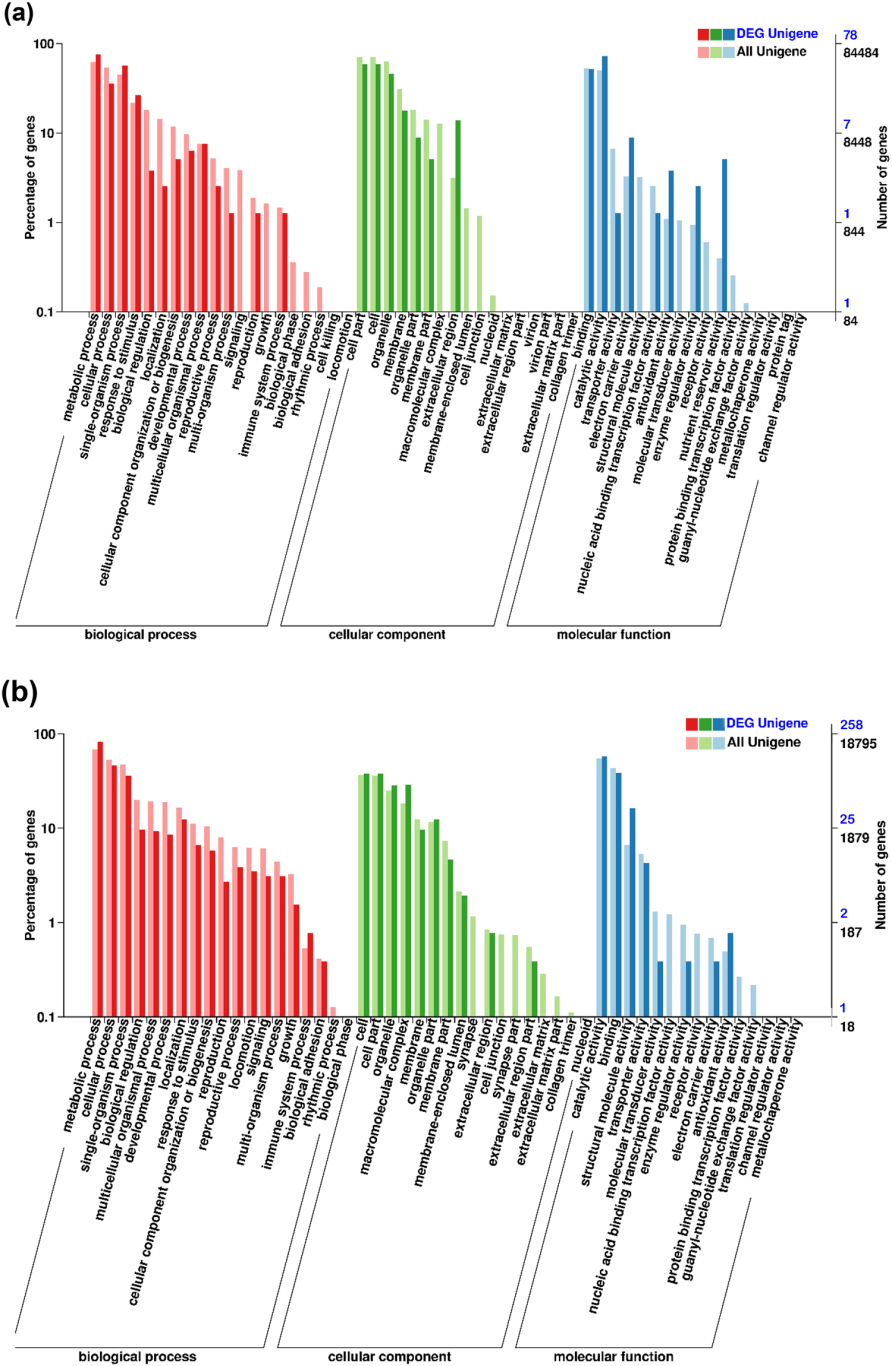
Gene Ontology (GO) categories of all unigenes and differentially expressed unigenes (DEG unigenes) in the wheat roots (**a**) and *Heterodera avenae* juveniles (**b**) in the study. The number and percentage of genes in each subcategory for the three main categories of biological process, cellular component, and molecular function are indicated for all the unigenes and DEG unigenes, respectively. On the right y-axis, blue and black numbers are DEG unigenes and all unigenes, respectively.

Based on the functional annotation obtained from GO enrichment analysis, the wheat DEGs were separated into 28 functional groups, which belong to three main categories: biological processes (60 DEGs), cellular components (54 DEGs), and molecular functions (68 DEGs) (Fig. 3a). In the biological process category, greater percentages of DEGs were involved in metabolic processes, single-organism processes and responses to stimuli compared to all unigenes of wheat roots. More proportions of DEGs in the cellular component category were localized to the extracellular region. The DEGs in the molecular function category were more enriched in GO class of nutrient reservoir activity, antioxidant activity, electron carrier activity, enzyme regulator activity and catalytic activity than all unigenes.

**Figure 4:**
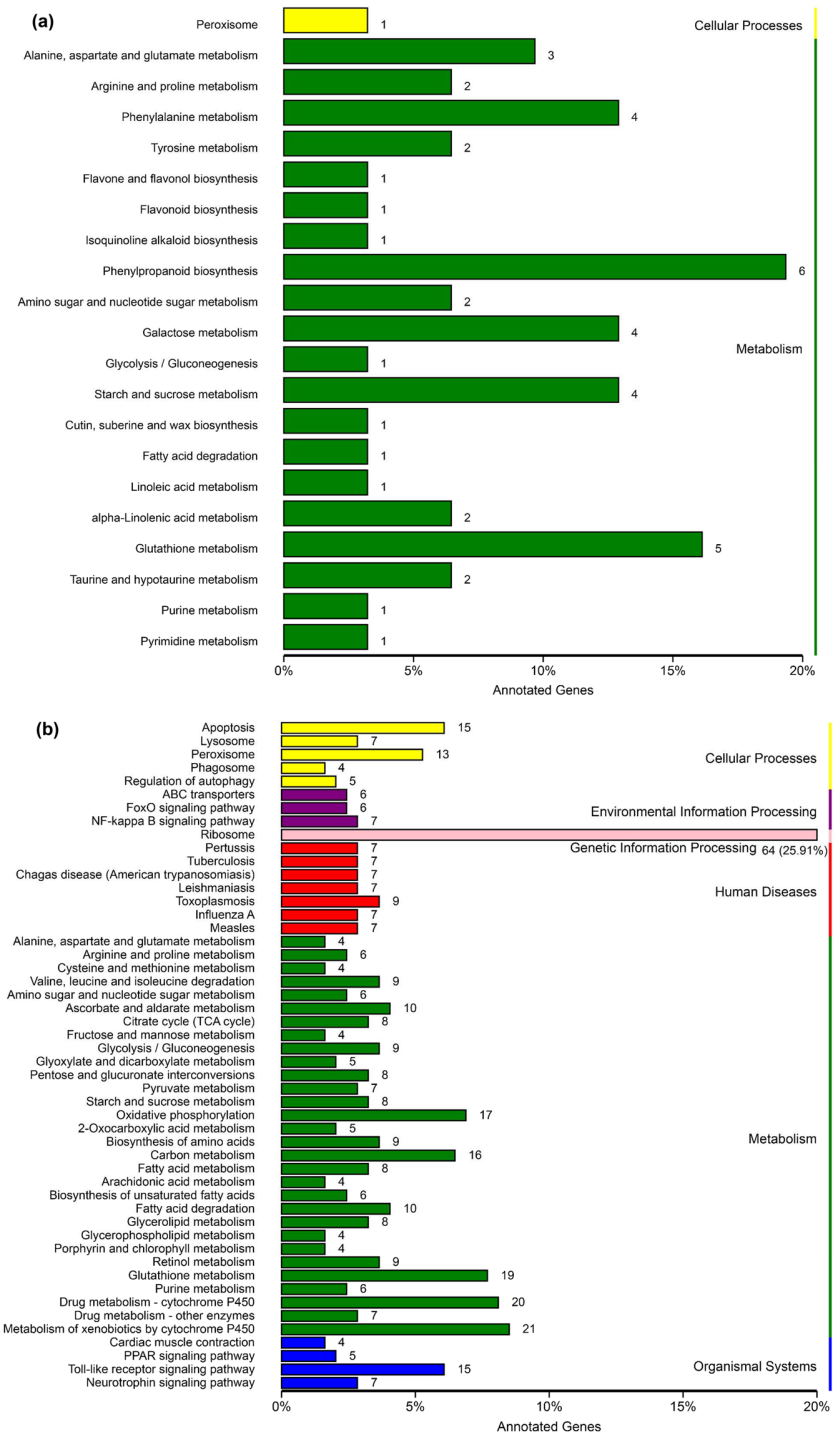
Kyoto Encyclopedia of Genes and Genomes (KEGG) enrichment analysis of differentially expressed genes (DEGs) of wheat roots (**a**) and *Heterodera avenae* juveniles (**b**) in the study. Cellular processes and metabolic pathways were enriched in wheat DEGs. Cellular processes, environmental information processing, genetic information processing, human diseases, metabolism and organismal system pathways were enriched in *H. avenae* DEGs. The x-axis shows the percentage of the annotated genes in each category and the number of genes is indicated at the top of the bar.

Analysis of KEGG pathways was performed to determine the biological functions of the DEGs. Thirty-one DEGs were allocated to 21 KEGG pathways (Fig. 4a, Supplementary Table S3). The phenylpropanoid biosynthesis pathway accounted for the highest number of DEGs, followed by glutathione metabolism, phenylalanine metabolism and starch and sucrose metabolism (Supplementary Table S3). Six DEGs, i.e., *Wheat_newGene_1897*, *gene:Traes_1AS_F9013A945*, *gene:Traes_2AS_EE549925C*, *gene:Traes_2DS_2CCCA54C1*, *gene:Traes_7DL_0D9D56EC9*, and *gene:Traes_7DL_4C9B51BF6*, were involved in the phenylpropanoid related pathways (phenylpropanoid biosynthesis or phenylalanine metabolism) (Supplementary Table S2). They were all down-regulated in the wheat roots that were exposed to the nematodes, and two of them, *gene:Traes_1AS_F9013A945* and *gene:Traes_2AS_EE549925C*, were validated by qPCR with similar expression patterns (Table 2).

**Figure 5:**
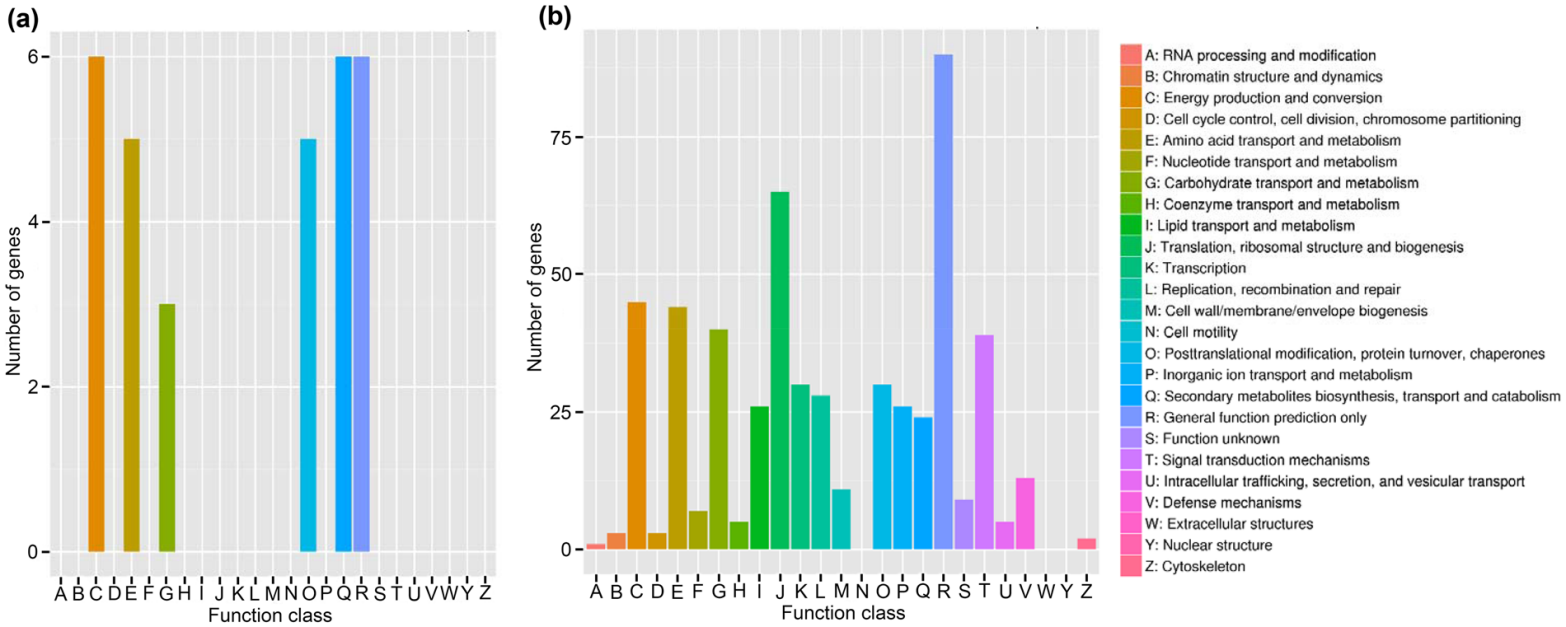
Clusters of Orthologous Groups (COG) function classification of differentially expressed genes (DEGs) of wheat roots (**a**) and *Heterodera avenae* juveniles (**b**) in the study. The y-axis shows the number of genes in each function class (in different colours on the x-axis).

Using COG annotation, 29 wheat DEGs were grouped into six COG functional classes, which included energy production and conversion; secondary metabolite biosynthesis, transport and catabolism; general function prediction only; posttranslational modification, protein turnover, chaperones; amino acid transport and metabolism; and carbohydrate transport and metabolism (Fig. 5a). All but four of these DEGs were down-regulated (Supplementary Table S2).

Among the DEGs of wheat roots, two protease inhibitor genes and one protease gene were annotated (Supplementary Table S2). Both *Wheat_newGene_1218* and *gene:Traes_1DL_A6553EC96* had GO annotation in the molecular function category of serine-type endopeptidase inhibitor activity (GO:0004867) and they were both localized in the cellular component of extracellular region (GO:0005576). Another DEG *Wheat_newGene_2674* was annotated as xylem cysteine proteinase 1 by Nr_annotation. These three DEGs were all down-regulated.

### Visualization of biotic stress pathways in the wheat DEGs

**Figure 6:**
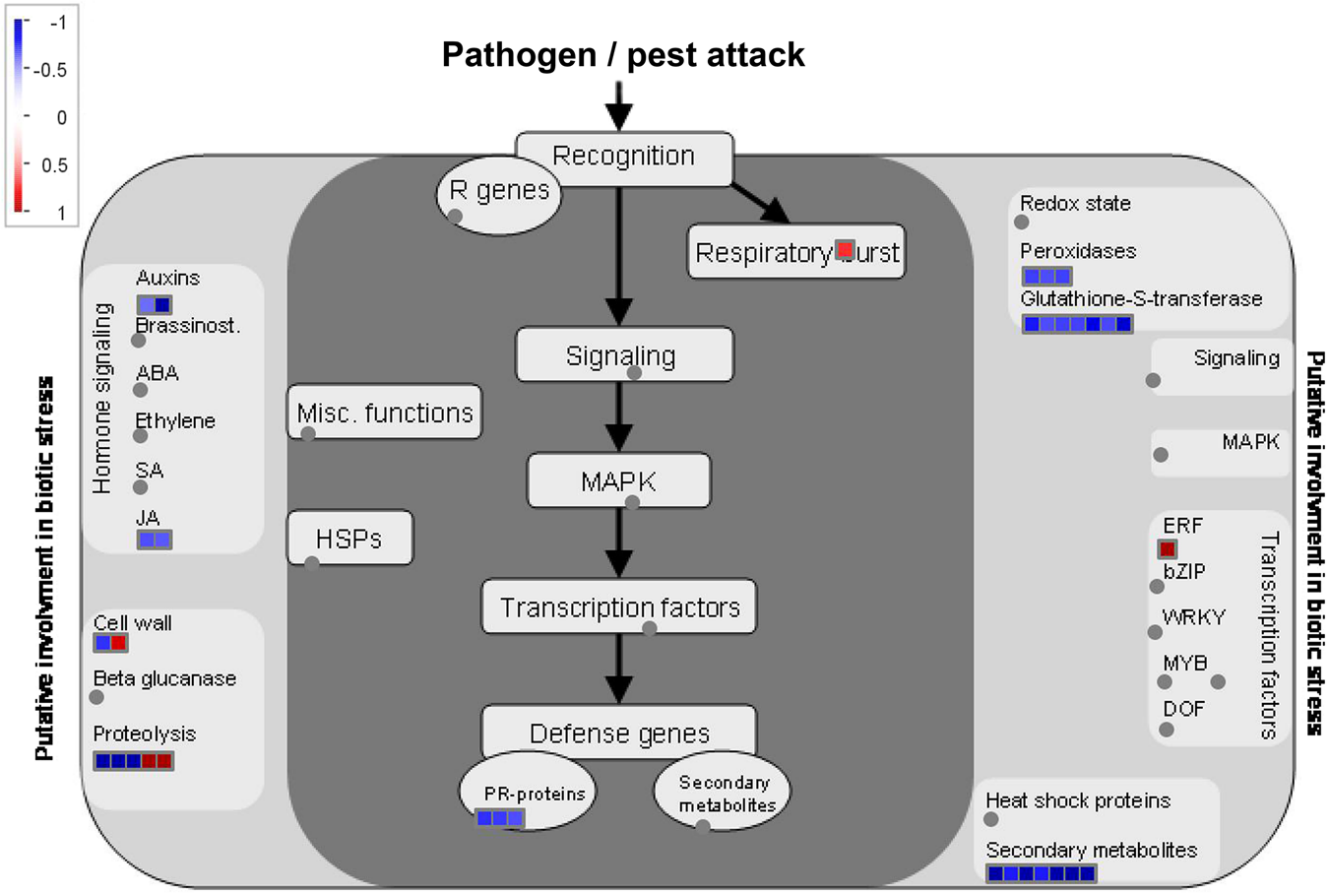
Visualization of biotic stress pathways in wheat differentially expressed genes (DEGs) by MapMan. Thirty-three data points showing putative involvement in biotic stress were mapped for 29 DEGs of wheat (see detailed information on the genes in Supplementary Table S6). Red and blue dots represent the genes that were up- and down-regulated in wheat roots when they were exposed to nematodes compared to the control (wheat roots without contact with the juvenile nematodes), respectively. The magnitude of the change in gene expression is associated with deepness of the colour.

A number of wheat DEGs were mapped to the biotic stress pathways as revealed by MapMan analysis (Fig. 6, Supplementary Table S4). Specifically, 33 data points that showed putative involvement in biotic stress were mapped for 29 wheat DEGs (Fig. 6), involving peroxidases, glutathione S transferases, hormone signaling (auxins and jasmonic acid), pathogenesis-related proteins, and secondary metabolites. Most of these DEGs were down-regulated in the biotic pathways (Fig. 6). This was confirmed by qPCR analysis of eight of those DEGs, as their expression patterns were consistent with that of mRNA-Seq in all cases but one (Table 2).

In the redox reaction, three DEGs (annotated as peroxidase 2, root peroxidase and class III peroxidase) and seven DEGs (annotated as putative glutathione-S-transferase, peroxidase and In2.1 protein) were mapped for peroxidases and glutathione-S-transferase, and they were all down-regulated, indicating weakening of the redox reaction. In the hormone signaling pathway, auxin and jasmonic acid (JA) signaling each contained two down-regulated DEGs, which were annotated as probable aldo-keto reductase 3, auxin-induced protein, lipoxygenase and 12-oxophytodienoate reductase, respectively. Seven data points mapped by the six down-regulated DEGs annotated as phenylalanine ammonialyase, agmatine coumaroyltransferase-2, tyrosine decarboxylase, leucoanthocyanidin dioxygenase, S-norcoclaurine synthase 1, flavonoid 3 & apos; 5 & apos;-hydroxylase 2 and S-norcoclaurine synthase 1 were almost involved in metabolism of phenylpropanoids and flavonoids. Defense genes coding for PR-proteins had three DEGs, which were all down-regulated with annotation as putative disease resistance RPP13-like protein and xylanase inhibitor. A down-regulated DEG (annotated as fasciclin-like protein FLA11) was mapped to cell wall protein and a predicted expansin-A13-like DEG up-regulated was related to cell wall modification pathway. The proteolysis pathway involved protease and ubiquitin were mapped by two down-regulated DEGs (annotated as xylem cysteine proteinase and ubiquitin) and two up-regulated DEGs (annotated as NEP1-interacting protein and E3 ubiquitin-protein ligase RING1-like). The respiratory burst involved one up-regulated DEG (annotated as respiratory burst oxidase homolog protein B). A transcription factor belongs to ethylene-responsive element binding protein family mapped by *gene:Traes_5DL_41E3B1B23* was an up-regulated DEG (annotated as ethylene-responsive transcription factor ERF071).

### Transcriptome data of CCN

Similar to the wheat roots, six samples of CCNs, from three replicates that were exposed to wheat roots and three for the controls without exposure to wheat roots, were separately subjected to RNA-sequencing analysis. The transcriptome analysis produced a total of 30.47 Gb clean data and ≥4.13 Gb per sample with the Quality Score of Q30 ≥ 89.1% (Supplementary Table S5). Altogether, 194,662 transcripts and 80,124 unigenes were obtained (Table 4). The total length, N50 length, and mean length of unigenes were 61,659,712 bp, 955 bp, and 769.55 bp, respectively (Table 4). A total of 15,197 unigenes were longer than 1 kb (Table 4). The proportions of mapped clean reads with the assembly data for each CCN sample ranged from 73.2% to 79.2%. A total of 43,741 unigenes were annotated according to the Nr, Swiss-Prot, GO, KEGG, COG, Clusters of Protein homology (KOG) and Pfam databases. Additionally, the replicates of CCNs showed good repeatability with each other (*r* ^2^ = 0.86~1.00) (Supplementary Fig. S1b).

**Table 4:** Summary of assembled transcripts and unigene data of *Heterodera avenae* in the study.

Length range	Transcripts	Unigenes
300–500 bp	54,351 (27.9)	41,355 (51.6)
500–1000 bp	44,713 (23.0)	23,572 (29.4)
1000–2000 bp	46,421 (23.9)	10,550 (13.2)
2000 + bp	49,177 (25.3)	4,647 (5.8)
Total number	194,662	80,124
Total length (bp)	295,450,379	61,659,712
N50 length (bp)	2,451	955
Mean length (bp)	1517.8	769.6

The number in brackets indicates the percentage of the transcripts or unigenes in that length range.

### CCN genes responding to wheat root attraction

The expression of genes of nematodes exposed to wheat roots was compared with the control (without exposure to wheat roots). A total of 879 unigenes were regarded as DEGs (FDR < 0.01 and FC ≥ 2). Most DEGs (867) were up-regulated and only 12 DEGs were down-regulated (Supplementary Fig. S2b, Supplementary Table S6). In addition, ten DEGs were analyzed by qPCR and their expression patterns were consistent with those of the mRNA-Seq results in all cases (Table 2). These results indicated that the CCNs were activated by the stimulation of wheat roots with up-regulation occurring for most of the genes detected. Results of functional annotation for the DEGs indicated that 742 of them (84.4%) were annotated in one or more of the Nr, Swiss-Prot, GO, KEGG, COG, KOG and Pfam databases (Table 3).

On the basis of their functional annotation, GO enrichment analysis classified 258 DEGs into 36 functional groups in three main categories: biological processes (159 DEGs), cellular components (107 DEGs), and molecular functions (223 DEGs) (Fig. 3b). The percentages of DEGs in GO class of metabolic processes and immune system processes were more than those of all unigenes of *H. avenae* juveniles. More proportions of DEGs in the cellular component category were localized to the macromolecular complex, organelle (part), and cell (part) compared to all the unigenes. The DEGs in the molecular function category were enriched more in the structural molecule activity, antioxidant activity and catalytic activity than all the unigenes.

Results of KEGG pathway analysis allocated 247 DEGs to 125 KEGG pathways (Supplementary Table S7), and 50 of the most significant pathways are shown in Fig. 4b. The pathways with the highest number of DEGs involved ribosomes (64 DEGs), and other pathways with a large numbers of DEGs included metabolism of xenobiotics by cytochrome P450, glutathione metabolism, and toll-like receptor signaling pathways (Supplementary Table S7). The ribosome pathway was more active in the responses of CCN exposed to and contacting with wheat roots than were the other pathways, which indicates that protein translation is much more active. Xenobiotics and drug metabolism were also considerably activated. The toll-like receptor signaling pathway (ko04620) involved 15 DEGs (Fig. 4b) and the DEGs related to IRAK1 (K04730; interleukin-1 receptor-associated kinase 1) and IRAK4 (K04733; interleukin-1 receptor-associated kinase 4) might lead to chemotactic effects (Supplementary Fig. S2).

A total of 386 DEGs were classified into 22 COG functional classes. The top four COG classes with the highest number of DEGs included those involved in general function prediction only (90 DEGs), translation, ribosomal structure and biogenesis (65 DEGs), energy production and conversion (45 DEGs), and amino acid transport and metabolism (44 DEGs) (Fig. 5b). The DEGs in those functional categories were the most strongly induced in the nematodes responding to exposure to the wheat roots.

### Effector prediction

**Figure 7:**
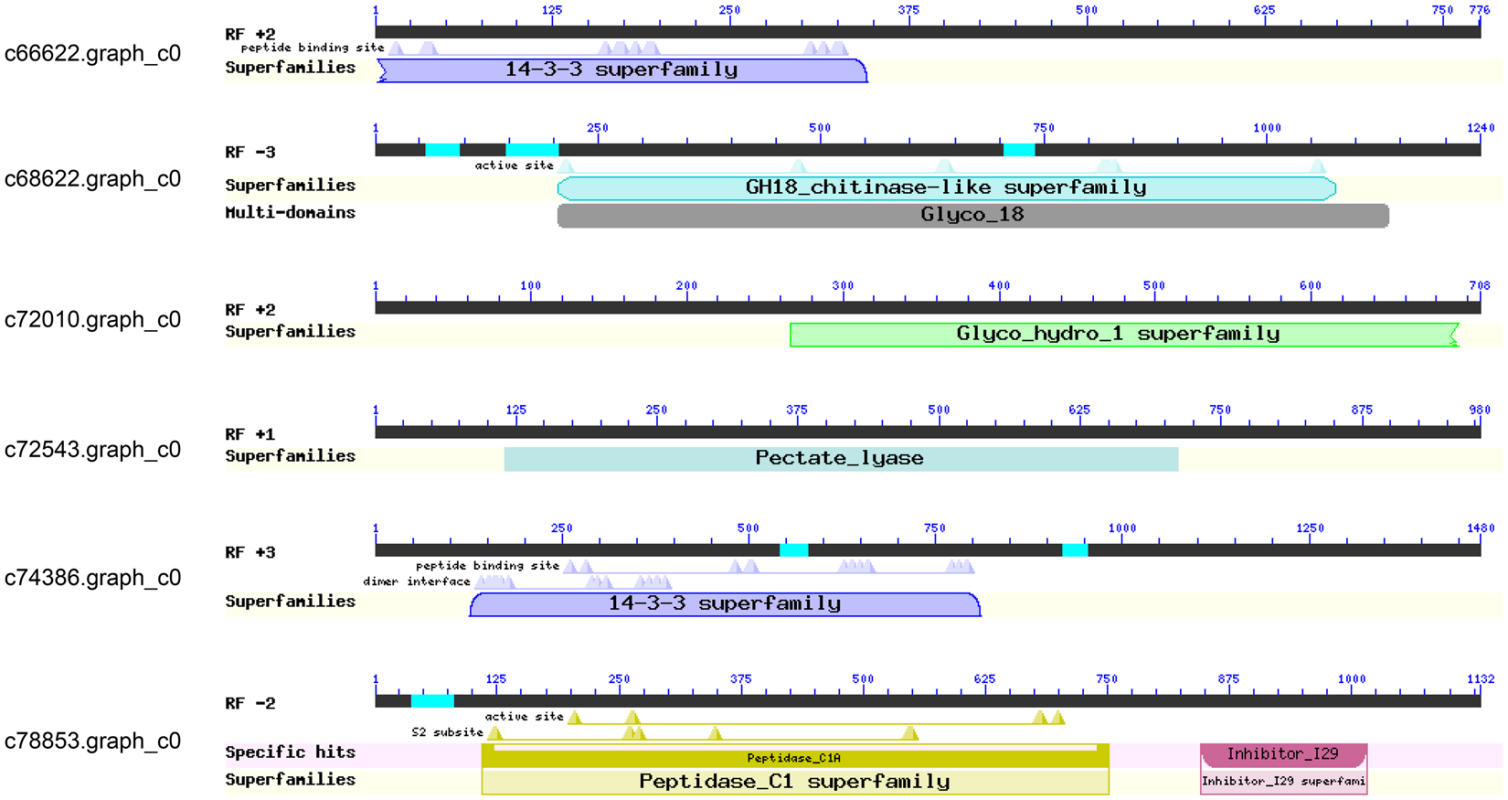
Structural domains of the six candidate effector genes of *Heterodera avenae* predicted through National Center for Biotechnology Information (NCBI). Both *c66622.graph_co* and *c74386.graph_c0* contain a putative 14-3-3 domain; *c68622.graph_c0* contains a GH18_chitinase-like domain or a glyco_18 domain; *c72010.graph_c0* contains a putative glyco_hydro_1 domain; *c72543.graph_c0* contains a putative pectate_lyase domain; and *c78853.graph_c0* contains a peptidase_C1 domain and an inhibitor_I29 domain (cathepsin propeptide inhibitor domain (I29)).

A total of 351 currently known effector gene sequences of PPNs were collected (Supplementary Table S8), and the DEGs of CCN were blasted against those sequences. Six DEGs were predicted to be homologous to the known effector genes 14-3-3, chitinase, beta-1,4-endoglucanase, pectate lyase, or cathepsin (Table 5). The description of their hit effector genes and the Nr annotations of the DEGs were consistent. The structural domains of those DEGs were predicted and they were also consistent with the gene descriptions (Fig. 7, Table 5). The DEGs that encode candidate effector proteins were all up-regulated when J2 nematodes were exposed to the wheat roots.

**Table 5:** Predicted effector genes mined from differentially expressed genes of *Heterodera avenae* when exposed to wheat roots.

Gene ID	Nr_annotation	Hit known effector genes	mRNA-Seq (log_2_FC)
c66622.graph_c0	PREDICTED: 14-3-3 protein epsilon-like [Mo]	GU130158 | 14-3-3 [Bx]	Inf (up)
**c68622.graph_c0**	Chitinase [Hg]	AF468679 | chitinase [Hg]	3.32
c72010.graph_c0	Beta-1,4-endoglucanase precursor [Gr]	AF006052 | Beta-1,4-endoglucanase-1 precursor [Hg]	1.02
**c72543.graph_c0**	Pectate lyase [Hg]	EF203898 | pectate lyase precursor [Hs]	1.94
c74386.graph_c0	14-3-3-like protein [Pp]	GU130158 | 14-3-3 [Bx]	6.27
**c78853.graph_c0**	Cathepsin L2 [Sc]	AJ557572 | putative cathepsin L protease [Mi]	1.83

Bx: *Bursaphelenchus xylophilus*, Gr: *Globodera rostochiensis*, Hg: *Heterodera glycines*, Hs: *Heterodera schachtii*, Mi: *Meloidogyne incognita*, Mo: *Metaseiulus occidentalis*, Pp: *Physarum polycephalum*, and Sc: *Sinonovacula constricta*. Known effector genes are shown as GenBank Accession numbers followed by gene description. Inf (up) indicated the expression of the gene was detected only in the treatment sample, but not in the control sample. The genes marked in bold were validated by qPCR (Table 2). FC, fold change (treatment versus control).

### Plant defence suppression by a predicted effector

**Figure 8:**
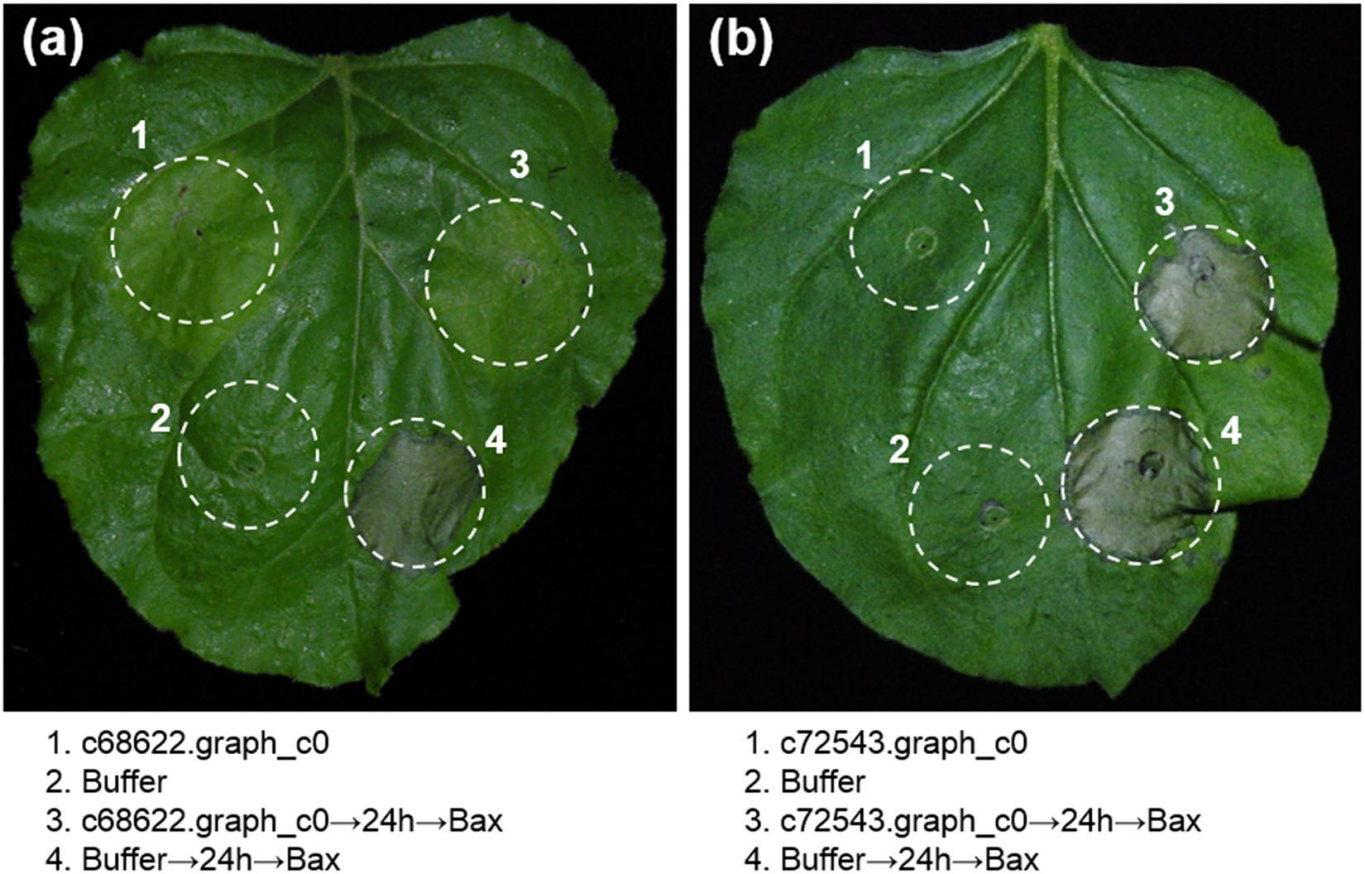
Assay for suppression of BAX-triggered cell death (BT-PCD) by the candidate *Heterodera avenae* effectors (**a**) c68622.graph_c0 and (**b**) c72543.graph_c0 in *Nicotiana benthamiana*. Leaves of *N. benthamiana* were infiltrated with the infiltration buffer or *Agrobacterium tumefaciens* cells containing a pGR107 vector carrying the candidate effector gene either alone or infiltration with *A. tumefaciens* cells carrying a mouse *Bax* gene 24 h later. Photos of the phenotypes of infiltrated *N. benthamiana* leaves were taken 6 days after infiltration. The spots with label 3 on the leaf show that the gene *c68622.graph_c0* suppressed the necrosis induced by *Bax*, but the gene *c72543.graph_c0* did not suppress necrosis.

Two putative effector genes *c68622.graph_c0* (hit chitinase [*H. glycines*]) and *c72543.graph_c0* (hit pectate lyase [*H. glycines*]) were selected for the BAX-triggered programmed cell death (BT-PCD) suppression assay in *Nicotiana benthamiana* Domin to verify their ability to suppress the plant defences. No obvious necrosis was observed on the infiltration spot of c68622.graph_c0 followed by BAX, while that of c72543.graph_c0 was obviously as necrotic as the infiltration buffer followed by BAX (Fig. 8). The two replicated results of the BT-PCD suppression assay in *N. benthamiana* were consistent. These findings suggest that the former gene suppresses BT-PCD while the latter gene does not. Therefore, *c68622.graph_c0* is a candidate effector gene from the *H. avenae* DEGs that may play a role in suppressing the plant’s defences.

## Discussion

The analysis of the DEGs provided an outline of the transcriptome responses of both wheat and *H. avenae* during the first step in the host-nematode interaction process (i.e., the contact stage), which has been largely overlooked in previous studies of the host-nematode interactions. Wheat is one of the most important crops in the world and cereal cyst nematodes are causing more and more damage to wheat crops^3^. Control of the nematodes before infection stage would be of great importance. This study focused on the contact stage of nematodes with wheat roots in an effort to investigate their early interaction mechanism. Because wheat has a reference genome while *H. avenae* does not, the transcriptome data of wheat was analyzed based on the reference genome and that of *H. avenae* was assembled and analyzed without a reference genome. With the following characteristics: well repeated biological replicates, high Quality Scores of Q30, well aligned clean reads of wheat with the reference genome, good quality of the assembled data of nematode and so on, the sequenced transcriptome data in the present study were believed to be reliable for the DEGs analysis. The mobile plant parasitic nematode *H. avenae* was shown to react much more actively with 879 DEGs, while the immobile wheat root tips only activated 93 DEGs. The response of the wheat root tips might have been induced by contact with the CCNs during their aggregation and/or some signal transduction occurred between the two organisms.

The GO annotation indicated that the DEGs induced in wheat and the CCN were mostly responsible for metabolism processes (Fig. 3). While the molecular functions of DEGs were divergent for the host and parasite. In the wheat host, nutrient reservoir, antioxidant activity, electron carrier activity and enzyme regulator activity were the top four enriched function categories of DEGs. In the CCN parasite, DEGs were enriched in the function categories of structural molecule activity and antioxidant activity. However, both wheat host and CCN parasite responded to each other with the DEGs enriched in the molecular function category of antioxidant activity. KEGG analysis of the wheat DEGs revealed that phenylpropanoid pathways associated with phenylpropanoid biosynthesis and phenylalanine metabolism were involved in the plant responses to nematode aggregation. It is increasingly clear that phenylpropanoid pathway is important in plant defence^38^. Previous research has demonstrated that the up-regulated components of phenylpropanoid pathway were also closely related to plant defence against infestations of nematodes, such as *G. rostochiensis*, *H. glycines*, and *H. avenae* ^31,39–41^. While the suppressed expression of genes in the phenylpropanoid pathway were related to susceptible reactions of plants to nematode infestation, for example, tomato to *G. rostochinensis* ^39^ and soybean to *H. glycines* ^42–44^. In the present study, components of this pathway were also transcriptionally regulated in the host responses to the aggregation of CCNs, indicating that plant defence might has already been affected when nematodes massed around plant roots (Fig. 4a). The wheat DEGs involved in this pathway were all down-regulated during the contact stage (Supplementary Table S2), which was consistent with previous studies on susceptible interactions during infection^39,40^. In addition, MapMan analysis revealed that a number of wheat DEGs were mapped in biotic stress pathways (Fig. 6, Supplementary Table S4). Those DEGs were mostly down-regulated indicating weakening of defense pathways. The DEGs in those pathways including redox reaction (involved peroxidase and glutathione-S-transferase), metabolism of phenylpropanoids and flavonoids, auxin and JA hormone signaling, and defense PR-proteins were all down-regulated. Modulation (up- or down-regulation) of peroxidase genes including class III peroxidase genes were previously shown to be involved in the CCN infestation of wheat^31,45^, while results of the current study showed that the three peroxidase genes were down-regulated during the contact stage.

Three DEGs related to protease or protease inhibitor genes were detected. The protease DEG *Wheat_newGene_2674* (annotated as xylem cysteine proteinase) was also involved in protein degradation of biotic stress pathway as revealed by MapMan analysis (Table S4). Cysteine protease was reported to be accumulated in maize vascular elements of leaf and root to defend against insect herbivores^46^. The xylem cysteine proteinase DEG in wheat roots was down-regulated and it might be related to weakening of wheat defense reaction to aggregation of nematodes. Protease inhibitors were involved in inducible defence in plants against herbivory including PPNs^47^. Serine protease inhibitors were also reported to confer resistance against nematode pests^48,49^. Two DEGs *Wheat_newGene_1218* and *gene:Traes_1DL_A6553EC96* annotated as serine protease inhibitors were down-regulated, indicating reduced defence of wheat at this stage.

The effector genes of PPNs have been reported to play important roles in their successful parasitism^50–52^, while it has not been reported whether the effector genes in CCN could be differentially expressed when nematodes approach the wheat roots. The results of transcriptome analysis identified six effector genes as DEGs from CCN during the contact stage and they were all up-regulated (Table 5), indicating that they were prepared in advance to promote subsequent infection by the nematodes. One candidate effector gene *c68622.graph_c0* (hit chitinase [*H. glycines*]) from the DEGs was shown to suppress BT-PCD, which triggers a process that physiologically resembles a hypersensitive defence response (Fig. 8). Many studies have demonstrated that the effectors of nematodes (including *H. avenae*) could affect plant defence as they did in other pathogens^53–56^. The up-regulation of the candidate effector gene *c68622.graph_c0* (hit chitinase [*H. glycines*]) during the contact stage as shown by both mRNA-Seq and qPCR analyses (Table 2) might be modulated by the nematodes in order to suppress plant immunity in the next infection stage. It appears that when they massed around wheat roots, the nematodes were armed already for fighting the plant defence to promote subsequent infection.

Other candidate effector genes in the *H. avenae* DEGs seemed to have different roles. For example, cell wall modification enzymes-like DEGs including *c72010.graph_c0* (Nr annotation as beta-1,4-endoglucanase precursor of *G. rostochiensis*) and *c72543.graph_c0* (Nr annotation as a pectate lyase of *H. glycines*) might degrade and soften the cell walls to allow the migration of PPNs inside the roots^46^. However, the DEGs for effectors perhaps were also effective during the host-parasite contact stage for signal induction or suppression in the host plant, as it has been reported that pre-parasitic J2s of RKN are stimulated to secrete quite a number of proteins, and could secrete low, but detectable levels of proteins even in the absence of stimulation^57,58^.

The phytohormones auxin and ethylene were reported to increase or decrease root attractiveness to nematodes, respectively^2,9–11^. During the contact stage of the nematodes with wheat roots after the attraction, some wheat genes related to phytohormones were also changed transcriptionally. For example, *gene:Traes_5DL_41E3B1B23* (annotation as an ethylene-responsive transcription factor ERF071 of *Arabidopsis thaliana*) was up-regulated and two auxin related genes *gene:Traes_2AL_1A870CE7B* (annotation as probable aldo-keto reductase 3) and *gene:Traes_6DS_768787FF4* (annotation as an auxin-induced protein) were down-regulated (Supplementary Table S4). Three DEGs related to flavonoid metabolism (*gene:TRAES3BF009500080CFD_g* annotated as leucoanthocyanidin dioxygenase, *gene:TRAES3BF168600 030CFD_g* annotated as S-norcoclaurine synthase 1, and *gene: Traes_2BL_37005C9E0* annotated as flavonoid 3 & apos;, 5 & apos;-hydroxylase 2) were also found to be down-regulated (Supplementary Table S4), while flavonoids can affect auxin level^59^. All the six DEGs were related to the phytohormones auxin or ethylene production, which indicates that phytohormones not only affect the attraction of nematodes to the wheat roots, but also are influenced by the aggregation of nematodes. Besides, down-regulation of auxin related genes and up-regulation of an ethylene related gene might reduce the attractiveness of wheat roots to nematodes as a response of the wheat roots to nematode aggregation. In addition, these six genes might also participate in plant defence as they were included in the biotic stress pathways by MapMan analysis (Supplementary Table S4).

In the current study, 15 DEGs of CCN were involved in the toll-like receptor signaling pathway (ko04620) (Fig. 4b). The DEGs related to IRAK1 and IRAK4 might influence chemotactic effects (Supplementary Fig. S2). Tropism of nematodes to wheat roots might involve this pathway. However, the functions of those genes in nematodes remain unknown.

## Methods

### Sampling and the attraction assay

Cysts of *H. avenae* (Ha43 pathotype group^60^) were collected from a CCN-infested field in Xingyang, Henan Province. Infective J2s were obtained by hatching the cysts at 15 °C for about one week following incubation for at least 8 weeks at 4 °C. Seeds of wheat cultivar Wenmai 19, which is susceptible to the Ha43 pathotype group of *H. avenae* ^61,62^, were germinated on moist filter paper in Petri dishes for 2 days at room temperature. A Pluronic F-127 gel (Sigma, Saint Louis, MO) (23%, w/v) was used as the medium for observing attraction of nematodes to the host roots^63^. In each biological replicate, five to six germinated wheat seeds with roots 2–3 cm long were transferred into a Petri dish (9 cm in diameter) containing 25,000~30,000 J2s suspended in 15 ml Pluronic F-127 gel for the attraction assay, which was defined as the treatment. The treatments with wheat seedlings or nematodes alone in the gel were used as the negative controls. After 3 h at room temperature, wheat root tips and nematodes in each Petri dish were collected and separately frozen in liquid nitrogen for RNA extraction. Simultaneously, wheat roots of the treatment were stained in a sodium hypochlorite-acid fuchsin solution^64^ to visualize any penetration of J2 nematodes into roots with a microscope (Olympus CX31, Tokyo, Japan).

Wheat seedlings were removed from the gel, rinsed in sterile-double-distilled water three times and dried on absorbent paper. Wheat root tips (*c*. 1 cm long) were then cut and collected in 2-ml RNase-off centrifuge tubes. Sterile-double-distilled water was added into the Petri dishes containing nematodes to liquidize the gels. The mixtures were poured into 50-ml centrifuge tubes and centrifuged at 6000 rpm for 2 min. Then, the nematodes at the bottom of the tubes were transferred to 1.5-ml centrifuge tubes and rinsed in sterile-double-distilled water containing 2 drops of 0.05% Tween-20 (polyoxyethylene (20) sorbitan monolaurate, Sinopharm Chemical Reagent, Beijing, China) three times (2000 rpm, 1 min). Finally, the nematodes were collected in 2-ml RNase-off centrifuge tubes.

### Bioinformatic analysis

Three replicates were included for each treatment or control. Total RNA was extracted from each sample of wheat roots or nematodes with the TRIzol reagent, which was used for library construction and mRNA-sequencing by HiSeq4000 (Biomarker Technologies Co. LTD, Beijing, China) independently. Data obtained from wheat roots and nematodes were analyzed by means of transcriptomic analysis with or without a reference genome (as *H. avenae* has no published reference genome).

For wheat, the raw reads of each sample were filtered to produce clean data, which were then aligned to the wheat genome using TopHat2^65^ (http://ftp.ensemblgenomes.org/pub/plants/release-30/fasta/triticum_aestivum/dna/). All the multiple mapped reads were allocated to specific gene on the reference genome by Cufflinks software^66^ using Maximum Likelihood Model. The abundance of transcripts was calculated as fragments per kilobase of transcript per million fragments mapped (FPKM)^67^ using the Cuffquant and Cuffnorm components in Cufflinks software^66^, and Pearson’s correlation coefficient *r* was calculated in R packages by its formula using the counts of all the transcriptomic data to compare repeats^68^. The DEGs between the control and the treatment sample were screened using DESeq^69^ with an FDR threshold < 0.05 and FC ≥ 1.5. To annotate the functions of the transcripts, the unigenes were blasted against the databases of Nr, Swiss-Prot, KEGG, and COG using BLAST program^70^ with an *E*-value ≤ 1e-5. The Blast2GO program was used to annotate the major GO categories of genes with an *E*-value ≤ 1e-5^71^. Furthermore, MapMan^72^ was used to visualize the biotic stress pathways of wheat DEGs after generating a mapping file of the wheat DEGs by Mercator^73^.

For CCN, the clean reads of each sample also were obtained after filtering, which were assembled together for all the nematode samples using Trinity software^74^. Clean data from each sample were aligned against the assembled transcripts or unigenes and the mapped reads were used for further analysis. The unigenes were annotated using BLAST program^70^ against Nr, Swiss-Prot, GO, KEGG, COG and KOG databases with an *E*-value ≤ 1e-5, and aligned with Pfam using HMMER^75^ with an *E*-value ≤ 10. To evaluate the expression levels (in the form of FPKM) of the unigenes, the reads of each sample were aligned with the assembled unigene database using Bowtie software^76^ and analyzed using RSEM program^77^. Repeat correlations were also tested by Pearson’s correlation coefficient^68^. The DEGs were identified by DESeq software^69^ with an FDR < 0.01 and FC ≥ 2.

The data described in this study have been deposited in the Gene Expression Omnibus^78^ of National Center for Biotechnology Information (NCBI) and are accessible through the GEO Series accession number GSE99228 (http://www.ncbi.nlm.nih.gov/geo/query/acc.cgi?acc = GSE99228).

### Validation of mRNA-Seq by qPCR

Based on their potential functional importance, 12 and 10 DEGs were selected for validation by qPCR from wheat and CCN, respectively. The cDNA was prepared from the remaining total RNA after transcriptome sequencing according to the instructions of the SuperScript™ III Reverse Transcriptase (Invitrogen, Carlsbad, CA, USA). A SYBR Green assay was used to quantify the expression of each gene using SYBR Premix Ex Taq (TaKaRa, Dalian, China) in a CFX Connect™ Real-Time PCR Detection System (Bio-Rad, Munich, Germany), with the primers for each DEG and the reference genes, *actin* for wheat^45^ and *GAPDH-1* for CCN^79^ (Supplementary Table S9). Three nematode or wheat biological replicates were analyzed for each gene with three technical replicates. Data were processed using the 2^−ΔΔCt^ method^80^ and analyzed statistically using the Student’s *t*-test in IBM SPSS Statistics 19 software (IBM Corp., Somers, NY, USA) to compare the difference between the treatments and the control samples at *P* < 0.05.

### *H. avenae* effector prediction

The sequences of 351 known parasitism effectors of PPNs (Supplementary Table S4) were collected and aligned with the *H. avenae* DEGs. Putative effector genes were obtained using an *E*-value ≤ 1e-5. Structural domain prediction of the genes was conducted by blasting the gene sequence at NCBI (http://blast.ncbi.nlm.nih.gov/Blast.cgi).

### Cell-death suppression assay in *N. benthamiana*

To detect plant defence suppression, two predicted effector genes from the *H. avenae* DEGs (i.e., *c68622.graph_c0* and *c72543.graph_c0*) were constructed in a PVX vector pGR107 (with the CP promoter) containing a flag-tag fused at the N-terminal, following the instructions of the In-Fusion HD Cloning Kit (Clontech, Palo Alto, CA, USA), with the primer pairs c68622-107f-S1/AS1 and c72543-107f-S1/AS1 (Supplementary Table S9). Construct of pGR107::Bax was provided by Dr. B.Y. Xie of the Chinese Academy of Agricultural Sciences, China. The constructs were verified by sequencing prior to transformation into *Agrobacterium tumefaciens* strain GV3101.

Plants of *N. benthamiana* were grown in a greenhouse for 4 to 6 weeks at 25 ± 2 °C under a photoperiod of 16 h light/8 h dark. An assay of the suppression of BT-PCD was carried out as described by Chen *et al*.^79^, except that 10 mM 2-(N-Morpholino) ethanesulfonic acid, 4-morpholineethanesulfonic acid (MES, pH 5.6) and 200 μM acetosyringone were added to 10 mM MgCl_2_ as the infiltration buffer. This assay was repeated twice, using 5 plant replicates in which three leaves were inoculated per plant.

### Data Availability

The datasets analyzed during the current study are available from the corresponding author on reasonable request.

## Electronic supplementary material

Supplementary Information_1

Dataset 1
