# Supplementary material for: Transcriptional responses of wheat and the cereal cyst nematode Heterodera avenae during their early contact stage
Source: Sci Rep. 2017 Nov 3;7:14471. doi: 10.1038/s41598-017-14047-y (PMC5670130; doi:10.1038/s41598-017-14047-y)
Supplement: Supplementary file 1 — Supplementary Information_1 [file 41598_2017_14047_MOESM1_ESM.doc]

Supplementary Information

**Transcriptional responses of wheat and the cereal cyst nematode *Heterodera avenae* during their early contact stage**

**Changlong Chen, Lei Cui, Yongpan Chen, Hongjun Zhang, Pei Liu, Peipei Wu, Dan Qiu, Jingwei Zou, Dan Yang, Li Yang, Hongwei Liu, Yang Zhou and Hongjie Li***

*** Correspondence:** Hongjie Li: [lihongjie@caas.cn](mailto:lihongjie@caas.cn)

The following supplementary materials are available for this article:

**Supplementary Figure S1.** The heatmap of repeat correlations of the transcriptomic data between each sample of wheat (a) and *Heterodera avenae* (b).

**Supplementary Figure S2.** Schematic of a toll-like receptor signaling pathway of cereal cyst nematode (CCN) exposed to wheat roots.

**Supplementary Table S1.** Number of sequenced data from each wheat root sample in the study.

**Supplementary Table S2.** Differentially expressed genes from wheat roots in the study (FDR < 0.05 and FC ≥ 1.5) (in an Excel file).

**Supplementary Table S3.** Kyoto Encyclopedia of Genes and Genomes (KEGG) pathways of differentially expressed genes (DEGs) of wheat roots in the study.

**Supplementary Table S4.** Differentially expressed genes (DEGs) from wheat roots involved in biotic stress pathways by MapMan visualization in the study.

**Supplementary Table S5.** Sequenced data of each *Heterodera avenae* sample in the study.

**Supplementary Table S6.** Differentially expressed genes of *Heterodera avenae* following exposure to wheat roots (FDR < 0.01 and FC ≥ 2) (in an Excel file).

**Supplementary Table S7.** Kyoto Encyclopedia of Genes and Genomes (KEGG) pathways of differentially expressed genes (DEGs) of *Heterodera avenae* when exposed to wheat roots.

**Supplementary Table S8.** The collected currently known putative effectors of plant parasitic nematodes (in an Excel file).

**Supplementary Table S9.** List of primers used in this study.

**Supplementary Note S1.** References of the collected currently known putative effectors from plant parasitic nematodes listed in Supplementary Table S8.

**Supplementary Figure 2.** The heatmap of repeat correlations of the transcriptomic data between each sample of wheat (a) and *Heterodera avenae* (b). The coloured grid between two samples means *r^2^* (*r*, Pearson’s correlation coefficient).

**
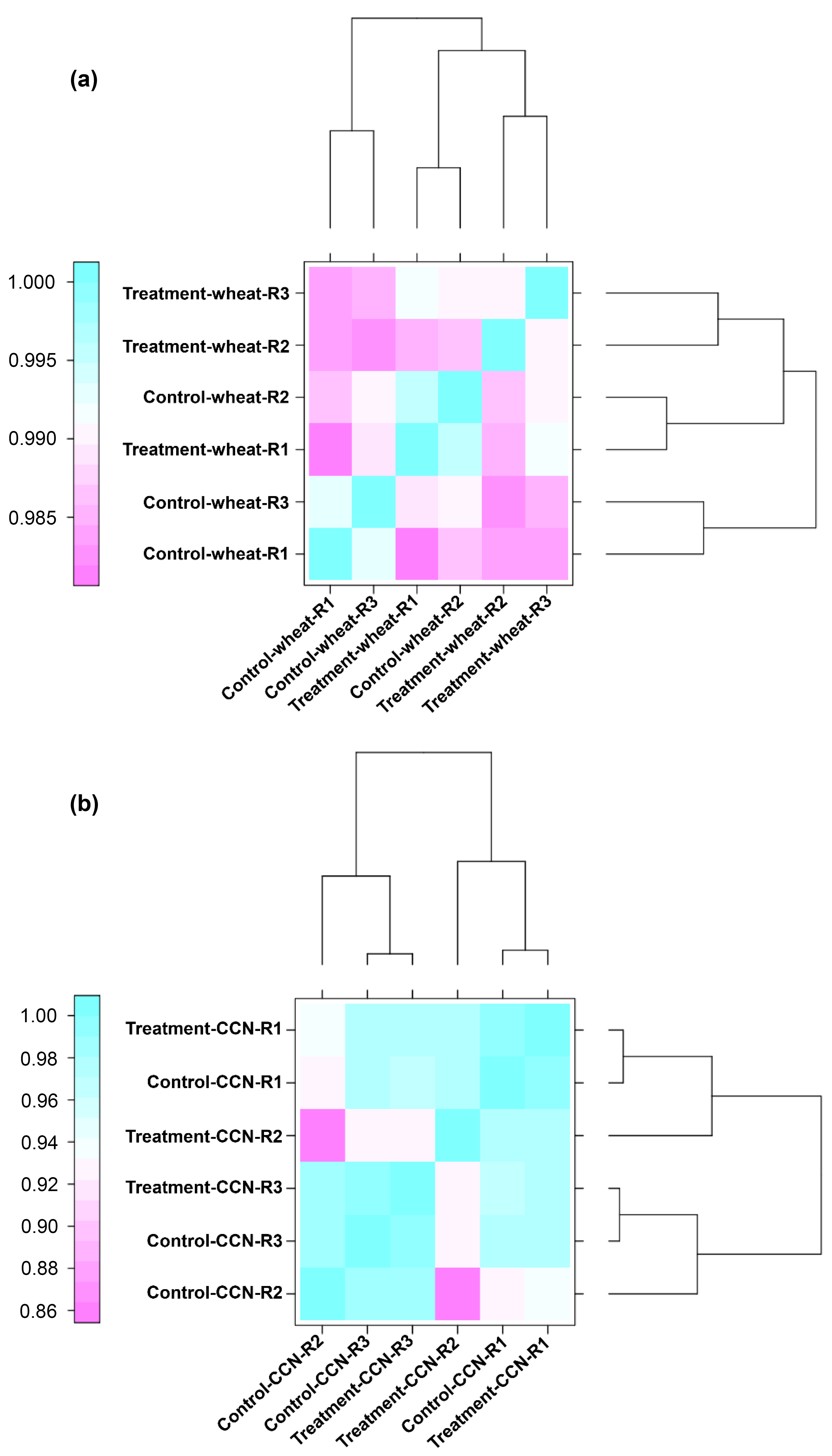
**

**Supplementary Figure 2.** Schematic of a toll-like receptor signaling pathway^80^ of cereal cyst nematode (CCN) exposed to wheat roots. Enzymes IRAK1 (interleukin-1 receptor-associated kinase 1) and IRAK4 (interleukin-1 receptor-associated kinase 4) in the red background are related to up-regulated genes, which could affect chemotactic effects (in the red circle).

**
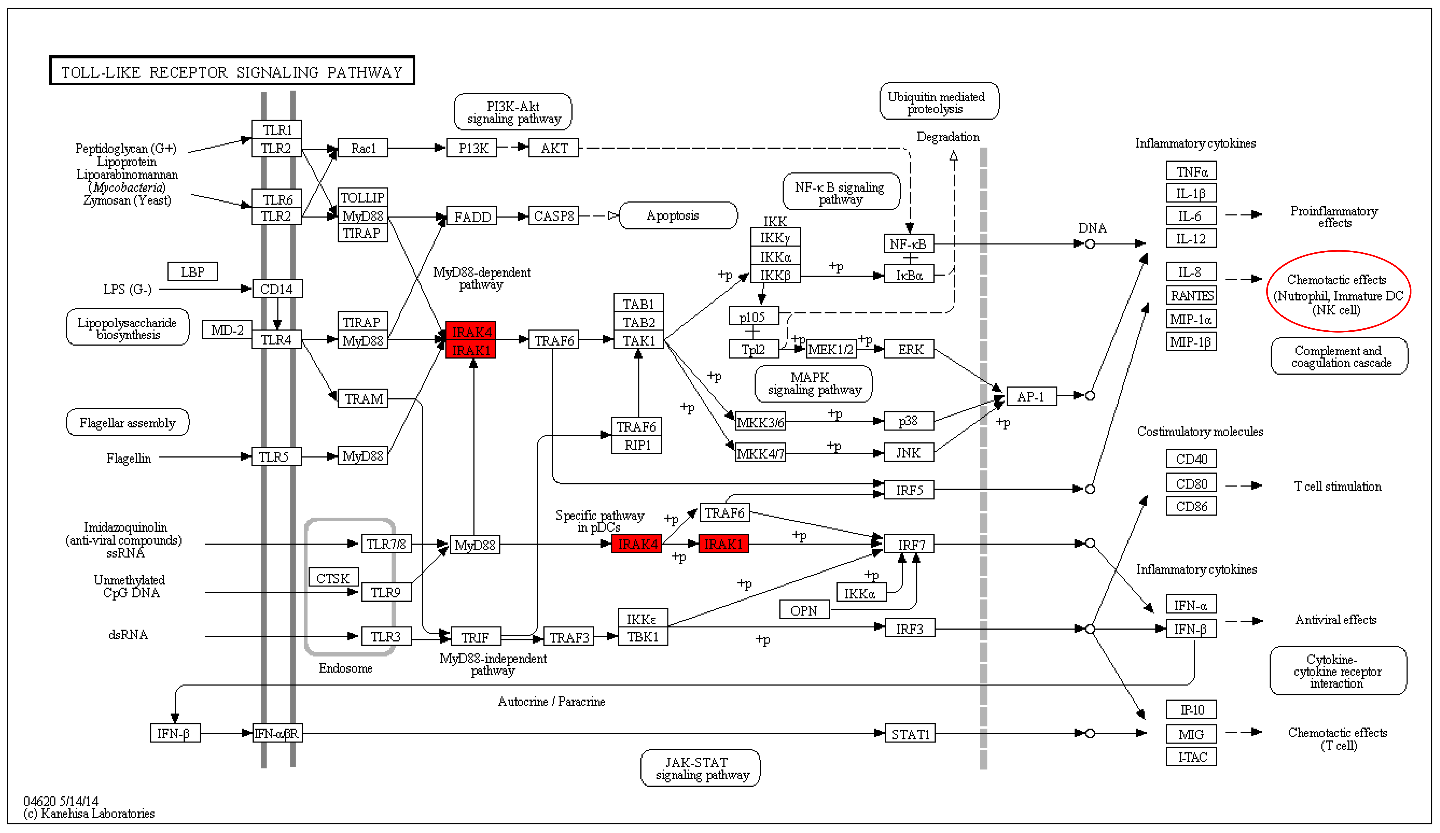
**

**Supplementary Table S1.** Number of sequenced data from each wheat root sample in the study.

| Samples | Raw reads | Clean reads | Clean bases | % ≥ Q30 |
| --- | --- | --- | --- | --- |
| Control-wheat-R1 | 28,795,253 | 28,751,614 | 8,525,607,812 | 90.76 |
| Control-wheat-R2 | 27,981,605 | 27,943,417 | 8,252,784,434 | 90.51 |
| Control-wheat-R3 | 29,080,776 | 29,038,972 | 8,597,818,322 | 90.43 |
| Treatment-wheat-R1 | 29,926,807 | 29,882,345 | 8,722,229,296 | 91.18 |
| Treatment-wheat-R2 | 30,037,750 | 29,993,761 | 8,860,788,048 | 90.60 |
| Treatment-wheat-R3 | 31,423,724 | 31,244,912 | 9,265,917,668 | 90.43 |

Q30 means that the accuracy of Base Calling is 99.9% in the sequencing, and the higher, the better.

**Supplementary Table S3. Kyoto Encyclopedia of Genes and Genomes (KEGG) pathways of differentially expressed genes (DEGs) of wheat roots in the study.**

| **KEGG pathway** | **ko ID** | **No. of DEGs** |
| --- | --- | --- |
| Taurine and hypotaurine metabolism | ko00430 | 2 |
| Phenylpropanoid biosynthesis | ko00940 | 6 |
| Purine metabolism | ko00230 | 1 |
| Tyrosine metabolism | ko00350 | 2 |
| Arginine and proline metabolism | ko00330 | 2 |
| Glycolysis/Gluconeogenesis | ko00010 | 1 |
| Cutin, suberine and wax biosynthesis | ko00073 | 1 |
| Starch and sucrose metabolism | ko00500 | 4 |
| Fatty acid degradation | ko00071 | 1 |
| Linoleic acid metabolism | ko00591 | 1 |
| Phenylalanine metabolism | ko00360 | 4 |
| Alanine, aspartate and glutamate metabolism | ko00250 | 3 |
| alpha-Linolenic acid metabolism | ko00592 | 2 |
| Pyrimidine metabolism | ko00240 | 1 |
| Flavone and flavonol biosynthesis | ko00944 | 1 |
| Amino sugar and nucleotide sugar metabolism | ko00520 | 2 |
| Peroxisome | ko04146 | 1 |
| Flavonoid biosynthesis | ko00941 | 1 |
| Isoquinoline alkaloid biosynthesis | ko00950 | 1 |
| Glutathione metabolism | ko00480 | 5 |
| Galactose metabolism | ko00052 | 4 |

**Supplementary Table S4. Differentially expressed genes (DEGs) from wheat roots involved in biotic stress pathways by MapMan visualization in the study.**

| **BINcode** | **BINname** | **Gene ID** | **Annotation** | **mRNA-Seq (log_2_FC)** |
| --- | --- | --- | --- | --- |
| 20.1.1 | Stress. biotic. respiratory burst | **gene:Traes_5BS_BCC1B9791** | Respiratory burst oxidase homolog protein B [*Oryza sativa* subsp. *japonica*] | 0.73 |
| 26.12 | Misc. peroxidases | Wheat_newGene_1897 | Peroxidase 2 [*Ae. tauschii*] | -0.70 |
| 26.12 | Misc. peroxidases | gene:Traes_2AS_EE549925C | Root peroxidase [*T. aestivum*] | -0.67 |
| 26.12 | Misc. peroxidases | gene:Traes_2DS_2CCCA54C1 | Class III peroxidase [*T. aestivum*] | -0.70 |
| 26.9 | Misc. glutathione S transferases | gene:Traes_1AL_CC4CF4E71 | Putative glutathione S-transferase GSTU6 [*T. urartu*] | -0.77 |
| 26.9 | Misc. glutathione S transferases | gene:Traes_2AS_EE549925C | Root peroxidase [*T. aestivum*] | -0.67 |
| 26.9 | Misc. glutathione S transferases | gene:Traes_2DS_2CCCA54C1 | Class III peroxidase [*T. aestivum*] | -0.70 |
| 26.9 | Misc. glutathione S transferases | gene:Traes_4AS_36CB7931F | Protein IN2-1 [*T. urartu*] | -0.69 |
| 26.9 | Misc. glutathione S transferases | gene:Traes_4BL_47A020E26 | Putative In2.1 protein [*T. aestivum*] | -0.87 |
| 26.9 | Misc. glutathione S transferases | gene:Traes_4DL_92CE4A8D1 | Putative In2.1 protein [*T. aestivum*] | -0.68 |
| 26.9 | Misc. glutathione S transferases | gene:Traes_5BL_B4E4DBF4A | Putative glutathione S-transferase GSTU1 [*T. urartu*] | -0.92 |
| 27.3.3 | RNA. regulation of transcription. AP2/EREBP, APETALA2/Ethylene-responsive element binding protein family | gene:Traes_5DL_41E3B1B23 | Ethylene-responsive transcription factor ERF071 [*Arabidopsis thaliana*] | 1.12 |
| 16.2.1.1 | Secondary metabolism. phenylpropanoids. lignin biosynthesis. PAL | **gene:Traes_1AS_F9013A945** | Phenylalanine ammonia-lyase [*Ae. tauschii*] | -1.17 |
| 16.2.1.4 | Secondary metabolism. phenylpropanoids. lignin biosynthesis. HCT | **gene:Traes_4BL_EB96605ED** | Agmatine coumaroyltransferase-2 [*H. vulgare*] | -0.76 |
| 16.4.1 | Secondary metabolism. N misc. alkaloid-like | gene:TRAES3BF264800010CFD_g | Tyrosine decarboxylase [*Papaver somniferum*] | -1.24 |
| 16.8.1 | Secondary metabolism. flavonoids. anthocyanins | gene:TRAES3BF009500080CFD_g | Leucoanthocyanidin dioxygenase [*Malus domestica*] | -0.76 |
| 16.8.3 | Secondary metabolism. flavonoids. dihydroflavonols | gene:TRAES3BF168600030CFD_g | S-norcoclaurine synthase 1 [*Coptis japonica*] | -1.00 |
| 16.8.3.3 | Secondary metabolism. flavonoids. dihydroflavonols. flavonoid 3''-monooxygenase | gene:Traes_2BL_37005C9E0 | Flavonoid 3 & apos;, 5 & apos;-hydroxylase 2 [*Petunia hybrida*] | -1.01 |
| 16.8.4 | Secondary metabolism. flavonoids. flavonols | gene:TRAES3BF168600030CFD_g | S-norcoclaurine synthase 1 [*Coptis japonica*] | -1.00 |
| 20.1.7 | Stress. biotic. PR-proteins | Wheat_newGene_14026 | Putative disease resistance RPP13-like protein 1 [*A. thaliana*] | -0.73 |
| 20.1.7 | Stress. biotic. PR-proteins | **gene:Traes_4AL_DD83F1A44** | xylanase inhibitor [*T. aestivum*] | -0.71 |
| 20.1.7 | Stress. biotic. PR-proteins | gene:Traes_4BS_FF3F5B3C51 | xylanase inhibitor [*T. aestivum*] | -0.67 |
| 17.2.3 | Hormone metabolism. auxin. induced-regulated-responsive-activated | **gene:Traes_2AL_1A870CE7B** | Probable aldo-keto reductase 3 [*O. sativa* subsp. *japonica*] | -0.61 |
| 17.2.3 | Hormone metabolism. auxin. induced-regulated-responsive-activated | **gene:Traes_6DS_768787FF4** | Auxin-induced protein [*Ae. tauschii*] | -1.31 |
| 17.7.1.2 | Hormone metabolism. jasmonate. synthesis-degradation. lipoxygenase | **gene:Traes_4BS_63DD9D036** | Lipoxygenase [*T. aestivum*] | -0.67 |
| 17.7.1.5 | Hormone metabolism. jasmonate. synthesis-degradation. 12-Oxo-PDA-reductase | gene:Traes_4BS_00408C6C6 | 12-oxophytodienoate reductase 1 [*A. thaliana*] | -0.65 |
| 10.5.1.1 | Cell wall. cell wall proteins. AGPs. AGP | gene:Traes_2BL_334D7D6C21 | fasciclin-like protein FLA11 [*T. aestivum*] | -0.72 |
| 10.7 | Cell wall. modification | gene:Traes_5DL_BCFA9DA27 | PREDICTED: expansin-A13-like [*Brachypodium distachyon*] | 0.90 |
| 29.5.3 | Protein. degradation. cysteine protease | Wheat_newGene_2674 | Xylem cysteine proteinase 1 [*Ae. tauschii*] | -1.24 |
| 29.5.11 | Protein. degradation. ubiquitin | Wheat_newGene_9066 | Ubiquitin (Precursor; Fragment) [*H. vulgare*] | -1.23 |
| 29.5.11.1 | Protein. degradation. ubiquitin. ubiquitin | Wheat_newGene_9066 | Ubiquitin (Precursor; Fragment) [*H. vulgare*] | -1.23 |
| 29.5.11.4.2 | Protein. degradation. ubiquitin. E3. RING | **gene:Traes_1BL_04B591073** | NEP1-interacting protein 2 [*A. thaliana*] | 1.07 |
| 29.5.11.4.2 | Protein. degradation. ubiquitin. E3. RING | gene:Traes_2DS_914822564 | E3 ubiquitin-protein ligase RING1-like [*A. thaliana*] | 1.18 |

BINcode and BINname, the classification of the gene in the MapMan system

FC, fold change (treatment vs. control)

The genes in bold were validated by qPCR (Table 2).

**Supplementary Table S5. Sequenced data of each *Heterodera avenae* sample in the study.**

| Sample | Clean read | Clean base | % ≥ Q30 | Mapped reads | Mapped ratio (%) |
| --- | --- | --- | --- | --- | --- |
| Control-CCN-R1 | 17,244,214 | 5,113,979,330 | 90.43 | 13,141,929 | 76.21 |
| Control-CCN-R2 | 13,883,955 | 4,131,325,432 | 89.97 | 10,770,197 | 77.57 |
| Control-CCN-R3 | 18,262,835 | 5,438,754,132 | 90.23 | 14,470,517 | 79.23 |
| Treatment-CCN-R1 | 16,478,523 | 4,820,620,244 | 91.31 | 12,057,690 | 73.17 |
| Treatment-CCN-R2 | 20,089,188 | 5,980,684,980 | 90.56 | 15,686,876 | 78.09 |
| Treatment-CCN-R3 | 16,758,335 | 4,986,601,212 | 89.09 | 13,000,783 | 77.58 |

Q30 means that the accuracy of Base Calling is 99.9% in the sequencing.

Mapped reads, number of clean reads mapped to the assembly unigene data.

Mapped ratio (%), ratio of mapped reads in the clean reads.

**Supplementary Table S7.** Kyoto Encyclopedia of Genes and Genomes (KEGG) pathways of differentially expressed genes (DEGs) of *Heterodera avenae* when exposed to wheat roots.

| KEGG pathway | ko_ID | No. of DEGs |
| --- | --- | --- |
| Taurine and hypotaurine metabolism | ko00430 | 2 |
| Valine, leucine and isoleucine biosynthesis | ko00290 | 1 |
| Adipocytokine signaling pathway | ko04920 | 1 |
| Toll-like receptor signaling pathway | ko04620 | 15 |
| Influenza A | ko05164 | 7 |
| Huntington's disease | ko05016 | 2 |
| Drug metabolism - cytochrome P450 | ko00982 | 20 |
| Lysosome | ko04142 | 7 |
| Fructose and mannose metabolism | ko00051 | 4 |
| 2-Oxocarboxylic acid metabolism | ko01210 | 5 |
| Adherens junction | ko04520 | 1 |
| Pentose and glucuronate interconversions | ko00040 | 8 |
| Apoptosis | ko04210 | 15 |
| Measles | ko05162 | 7 |
| Drug metabolism - other enzymes | ko00983 | 7 |
| Sulfur metabolism | ko00920 | 3 |
| ECM-receptor interaction | ko04512 | 1 |
| Insect hormone biosynthesis | ko00981 | 1 |
| Selenocompound metabolism | ko00450 | 2 |
| Glyoxylate and dicarboxylate metabolism | ko00630 | 5 |
| Glutathione metabolism | ko00480 | 19 |
| Arginine and proline metabolism | ko00330 | 6 |
| Leishmaniasis | ko05140 | 7 |
| Galactose metabolism | ko00052 | 3 |
| Degradation of aromatic compounds | ko01220 | 1 |
| D-glutamine and D-glutamate metabolism | ko00471 | 1 |
| Regulation of actin cytoskeleton | ko04810 | 1 |
| beta-Alanine metabolism | ko00410 | 4 |
| N-glycan biosynthesis | ko00510 | 1 |
| Phenylalanine metabolism | ko00360 | 1 |
| Pentose phosphate pathway | ko00030 | 3 |
| Alzheimer's disease | ko05010 | 2 |
| SNARE interactions in vesicular transport | ko04130 | 1 |
| Focal adhesion | ko04510 | 1 |
| NF-kappa B signaling pathway | ko04064 | 7 |
| Pyrimidine metabolism | ko00240 | 4 |
| Melanogenesis | ko04916 | 1 |
| Alanine, aspartate and glutamate metabolism | ko00250 | 4 |
| Primary bile acid biosynthesis | ko00120 | 2 |
| ABC transporters | ko02010 | 6 |
| mRNA surveillance pathway | ko03015 | 2 |
| Glycine, serine and threonine metabolism | ko00260 | 3 |
| Steroid hormone biosynthesis | ko00140 | 2 |
| Hedgehog signaling pathway | ko04340 | 3 |
| Biosynthesis of unsaturated fatty acids | ko01040 | 6 |
| alpha-linolenic acid metabolism | ko00592 | 4 |
| MAPK signaling pathway | ko04010 | 1 |
| Glycerolipid metabolism | ko00561 | 8 |
| Peroxisome | ko04146 | 13 |
| Pyruvate metabolism | ko00620 | 7 |
| Glycolysis/gluconeogenesis | ko00010 | 9 |
| Adrenergic signaling in cardiomyocytes | ko04261 | 1 |
| FoxO signaling pathway | ko04068 | 6 |
| Ribosome biogenesis in eukaryotes | ko03008 | 1 |
| Wnt signaling pathway | ko04310 | 3 |
| RNA transport | ko03013 | 4 |
| Parkinson's disease | ko05012 | 2 |
| Complement and coagulation cascades | ko04610 | 1 |
| Endocytosis | ko04144 | 1 |
| Insulin signaling pathway | ko04910 | 1 |
| mTOR signaling pathway | ko04150 | 3 |
| Amino sugar and nucleotide sugar metabolism | ko00520 | 6 |
| Cardiac muscle contraction | ko04260 | 4 |
| Inositol phosphate metabolism | ko00562 | 2 |
| Starch and sucrose metabolism | ko00500 | 8 |
| Progesterone-mediated oocyte maturation | ko04914 | 1 |
| p53 signaling pathway | ko04115 | 1 |
| Toxoplasmosis | ko05145 | 9 |
| Regulation of autophagy | ko04140 | 5 |
| Cyanoamino acid metabolism | ko00460 | 3 |
| Neurotrophin signaling pathway | ko04722 | 7 |
| Ubiquitin mediated proteolysis | ko04120 | 1 |
| Tryptophan metabolism | ko00380 | 4 |
| PPAR signaling pathway | ko03320 | 5 |
| Citrate cycle (TCA cycle) | ko00020 | 8 |
| Histidine metabolism | ko00340 | 3 |
| Spliceosome | ko03040 | 1 |
| Purine metabolism | ko00230 | 6 |
| Carbon metabolism | ko01200 | 16 |
| Steroid biosynthesis | ko00100 | 1 |
| Fatty acid biosynthesis | ko00061 | 2 |
| Butanoate metabolism | ko00650 | 1 |
| Sphingolipid metabolism | ko00600 | 2 |
| TGF-beta signaling pathway | ko04350 | 1 |
| Nitrogen metabolism | ko00910 | 1 |
| AMPK signaling pathway | ko04152 | 1 |
| Ascorbate and aldarate metabolism | ko00053 | 10 |
| Biosynthesis of amino acids | ko01230 | 9 |
| MAPK signaling pathway – fly | ko04013 | 1 |
| Glycosaminoglycan biosynthesis - heparan sulfate / heparin | ko00534 | 1 |
| Fatty acid metabolism | ko01212 | 8 |
| Fatty acid degradation | ko00071 | 10 |
| Protein export | ko03060 | 3 |
| GABAergic synapse | ko04727 | 1 |
| Tight junction | ko04530 | 2 |
| Other types of O-glycan biosynthesis | ko00514 | 1 |
| Phosphatidylinositol signaling system | ko04070 | 2 |
| Gap junction | ko04540 | 1 |
| Propanoate metabolism | ko00640 | 2 |
| Valine, leucine and isoleucine degradation | ko00280 | 9 |
| RNA degradation | ko03018 | 1 |
| Cysteine and methionine metabolism | ko00270 | 4 |
| Pantothenate and CoA biosynthesis | ko00770 | 1 |
| Retinol metabolism | ko00830 | 9 |
| Protein processing in endoplasmic reticulum | ko04141 | 3 |
| Antigen processing and presentation | ko04612 | 1 |
| Pertussis | ko05133 | 7 |
| Vascular smooth muscle contraction | ko04270 | 1 |
| Oxidative phosphorylation | ko00190 | 17 |
| Chagas disease (American trypanosomiasis) | ko05142 | 7 |
| Phagosome | ko04145 | 4 |
| Glycerophospholipid metabolism | ko00564 | 4 |
| Linoleic acid metabolism | ko00591 | 1 |
| One carbon pool by folate | ko00670 | 1 |
| Non-alcoholic fatty liver disease (NAFLD) | ko04932 | 1 |
| Arachidonic acid metabolism | ko00590 | 4 |
| Platelet activation | ko04611 | 1 |
| Tyrosine metabolism | ko00350 | 2 |
| Tuberculosis | ko05152 | 7 |
| Porphyrin and chlorophyll metabolism | ko00860 | 4 |
| Metabolism of xenobiotics by cytochrome P450 | ko00980 | 21 |
| Lysine degradation | ko00310 | 3 |
| Caffeine metabolism | ko00232 | 2 |
| Type I diabetes mellitus | ko04940 | 1 |
| Ribosome | ko03010 | 64 |

**Supplementary Table S9.** List of primers used in this study.

| Gene ID | Name | Sequence (5ꞌ-3ꞌ) |
| --- | --- | --- |
| **For PVX vector construct** | | |
| *c68622.graph_c0* | c68622-107f-S1 | TAGTGGATCCCCCGGGATGAAAAACTTTTTGGGC |
|  | c68622-107f-AS1 | TTCATCGGCGGTCGACCTACGACATCGAAGAAG |
| *c72543.graph_c0* | c72543-107f-S1 | TAGTGGATCCCCCGGGATGCATTCGTTCCTC |
|  | c72543-107f-AS1 | TTCATCGGCGGTCGACTTAGCCCATTTTTGTGC |
| **For wheat qPCR** | | |
| *gene:TRAES3BF074000020CFD_g* | Ta4-qS1 | TCATCCACCGCCACATT |
|  | Ta4-qAS1 | TGAGCCCTGGGACAAAGT |
| *gene:Traes_1AS_F9013A945* | Ta5-qS1 | TTGACCCACAAACTGAAGCA |
|  | Ta5-qAS1 | TGGCAGCACGGATGACCT |
| *gene:Traes_2AL_8394449B2* | Ta7-qS1 | GACTCCAGAGCAGCAGAACA |
|  | Ta7-qAS1 | ACTCCGTGCCGTCATCCTT |
| *gene:Traes_2AS_EE549925C* | Ta8-qS1 | TTCCACGACTGCTTTGT |
|  | Ta8-qAS1 | AGGGAGGTCACTGTTC |
| *gene:Traes_3DL_EE0699FDC* | Ta11-qS1 | CTACGCTGAAGGTGCCA |
|  | Ta11-qAS1 | GCCATTCTCATCTGTGCC |
| *gene:Traes_6DS_768787FF4* | Ta15-qS1 | CTCCGCTTCCACCATAA |
|  | Ta15-qAS1 | TGTGCTCCAGGTTCTCG |
| *gene:Traes_5BS_BCC1B9791* | Ta16-qS1 | CATCATGGTGGTGCTCATGG |
|  | Ta16-qAS1 | CCGGTTGATGTAGAGGCAGA |
| *gene:Traes_4BL_EB96605ED* | Ta18-qS1 | GAACAGGTACAAGTGCGGTG |
|  | Ta18-qAS1 | CGTGGTGAAGTCCTTACCCT |
| *gene:Traes_4AL_DD83F1A44* | Ta19-qS1 | CAACTACCACCTCGACCTGT |
|  | Ta19-qAS1 | AGGATGTTCTTGGACTGGCA |
| *gene:Traes_2AL_1A870CE7B* | Ta21-qS1 | CTCTACTACCAGCACCGCAT |
|  | Ta21-qAS1 | CCGACAACCCGACGTATTTC |
| *gene:Traes_4BS_63DD9D036* | Ta22-qS1 | GAGCCGTTTGTGATCTCGAC |
|  | Ta22-qAS1 | CTCCGTGAACTTCCAGTCCT |
| *gene:Traes_1BL_04B591073* | Ta25-qS1 | GACATCATCCTCAGCCTGCT |
|  | Ta25-qAS1 | GCGCTTATCTGACTCTGCAC |
| *actin* | Taactin-qS1 | GAAGCTGCAGGTATCCATGAGACC |
|  | Taactin-qAS1 | AGGCAGTGATCTCCTTGCTCATC |
| **For *Heterodera avenae* qPCR** | | |
|  |  |  |
| *c73395.graph_c0* | Ha1-qS1 | CGTGCGTTGGGATTTGA |
|  | Ha1-qAS1 | CAGCCAGCAGTTGGGTATT |
| *c62312.graph_c0* | Ha2-qS1 | ACCAACATCGCATTCACTCA |
|  | Ha2-qAS1 | GGGTAAGAAAGAAACGCATAAACA |
| *c73973.graph_c0* | Ha3-qS1 | CGTTCGCTGTTCCTTCT |
|  | Ha3-qAS1 | AGCAGTGGCATTATCTCA |
| *c78521.graph_c0* | Ha4-qS1 | GCCCATAAATCGTCATAGTTG |
|  | Ha4-qAS1 | CTCGTGCGTTTCTTTCTCAT |
| *c54125.graph_c0* | Ha6-qS1 | TTAGCGGACGAAACAACG |
|  | Ha6-qAS1 | AAGAGGAGGTAAATAAGAACAC |
| *c72543.graph_c0* | Ha7-qS1 | CAGTGACGGCAACAATCCA |
|  | Ha7-qAS1 | CATCTTCGCCCACCTTTT |
| *c76930.graph_c0* | Ha8-qS1 | GGGGAACCCAGCCATCA |
|  | Ha8-qAS1 | TCGTGCTCCGTCCTACTCG |
| *c79218.graph_c0* | Ha9-qS1 | GCAAAGAAGGGCAAGGA |
|  | Ha9-qAS1 | CAAACCCGCCAATCACA |
| *c68622.graph_c0* | Ha11-qS1 | CAACACCGTCCAACTCA |
|  | Ha11-qAS1 | GGGCAAATCCGCTAACA |
| *c78853.graph_c0* | Ha17-qS1 | TGCGTTTGTTACGGGTCA |
|  | Ha17-qAS1 | CTCGCTGTTGGTTATGG |
| *GAPDH-1* | GAPDH-qS1 | AGCGGCACAGAACATCATCC |
|  | GAPDH-qAS1 | GGTCCTCCGTGTAGCCCAAA |

**Supplementary Note S1.** References of the collected currently known putative effectors from plant parasitic nematodes listed in Supplementary Table S8.

1. Lilley, C. J., Goodchild, S. A., Atkinson, H. J. & Urwin, P. E. Cloning and characterisation of a *Heterodera glycines* aminopeptidase cDNA. *Int. J. Parasitol.* **35**, 1577-1585 (2005).

2. Jones, J. T. *et al*. Identification and functional characterization of effectors in expressed sequence tags from various life cycle stages of the potato cyst nematode *Globodera pallida*. *Mol. Plant Pathol.* **10**, 815-828 (2009).

3. Jacob, J., Mitreva, M., Vanholme, B. & Gheysen, G. Exploring the transcriptome of the burrowing nematode *Radopholus similis*. *Mol. Genet. Genomics* **280**, 1-17 (2008).

4. Urwin, P. E., Lilley, C. J., McPherson, M. J. & Atkinson, H. J. Characterization of two cDNAs encoding cysteine proteinases from the soybean cyst nematode *Heterodera glycines*. *Parasitology* **114**, 605-613 (1997).

5. Gao, B. *et al*. Identification of putative parasitism genes expressed in the esophageal gland cells of the soybean cyst nematode *Heterodera glycines*. *Mol. Plant-Microbe Interact.* **14**, 1247-1254 (2001).

6. Dautova, M. *et al*. Single pass cDNA sequencing - a powerful tool to analyse gene expression in preparasitic juveniles of the southern root-knot nematode *Meloidogyne incognita*. *Nematology* **3**, 129-139 (2001).

7. Shingles, J., Lilley, C. J., Atkinson, H. J. & Urwin, P. E. *Meloidogyne incognita*: molecular and biochemical characterisation of a cathepsin L cysteine proteinase and the effect on parasitism following RNAi. *Exp. Parasitol.* **115**, 114-120 (2007).

8. Robertson, L., Robertson, W. M. & Jones, J. T. Direct analysis of the secretions of the potato cyst nematode *Globodera rostochiensis*. *Parasitology* **119**, 167-176 (1999).

9. Furlanetto, C., Cardle, L., Brown, D. J. F. & Jones, J. T. Analysis of expressed sequence tags from the ectoparasitic nematode *Xiphinema index*. *Nematology* **7**, 95-104 (2005).

10. Vieira, P. *et al*. The plant apoplasm is an important recipient compartment for nematode secreted proteins. *J. Exp. Bot.* **62**, 1241-1253 (2011).

11. Haegeman, A., Kyndt, T. & Gheysen, G. The role of pseudo-endoglucanases in the evolution of nematode cell wall-modifying proteins. *J. Mol. Evol.* **70**, 441-452 (2010).

12. Uehara, T., Kushida, A. & Momota, Y. PCR-based cloning of two beta-1,4-endoglucanases from the root-lesion nematode *Pratylenchus penetrans*. *Nematology* **3**, 335-341 (2001).

13. Smant, G. *et al*. Endogenous cellulases in animals: isolation of beta-1, 4-endoglucanase genes from two species of plant-parasitic cyst nematodes. *Proc. Natl. Acad. Sci. USA* **95**, 4906-4911 (1998).

14. Yan, Y. *et al*. Genomic organization of four beta-1,4-endoglucanase genes in plant-parasitic cyst nematodes and its evolutionary implications. *Gene* **220**, 61-70 (1998).

15. Rosso, M. N. *et al*. Isolation of a cDNA encoding a beta-1,4-endoglucanase in the root-knot nematode *Meloidogyne incognita* and expression analysis during plant parasitism. *Mol. Plant-Microbe Interact.* **12**, 585-591 (1999).

16. Ledger, T. N., Jaubert, S., Bosselut, N., Abad, P. & Rosso, M. N. Characterization of a new beta-1,4-endoglucanase gene from the root-knot nematode *Meloidogyne incognita* and evolutionary scheme for phytonematode family 5 glycosyl hydrolases. *Gene* **382**, 121-128 (2006).

17. Goellner, M., Smant, G., de Boer, J. M., Baum, T. J. & Davis, E. L. Isolation of beta-1,4-endoglucanase genes from *Globodera tabacum* and their expression during parasitism. *J. Nematol.* **32**, 154-165 (2000).

18. de Meutter, J., Vanholme, B., Bauw, G., Tytgat, T. & Gheysen, G. Preparation and sequencing of secreted proteins from the pharyngeal glands of the plant parasitic nematode *Heterodera schachtii*. *Mol. Plant Pathol.* **2**, 297-301 (2001).

19. Haegeman, A., Jacob, J., Vanholme, B., Kyndt, T. & Gheysen, G. A family of GHF5 endo-1,4-beta-glucanases in the migratory plant-parasitic nematode *Radopholus similis*. *Plant Pathol.* **57**, 581-590 (2008).

20. Rehman, S. *et al*. Identification and characterization of the most abundant cellulases in stylet secretions from *Globodera rostochiensis*. *Phytopathology* **99**, 194-202 (2009).

21. Kikuchi, T., Jones, J. T., Aikawa, T., Kosaka, H. & Ogura, N. A family of glycosyl hydrolase family 45 cellulases from the pine wood nematode *Bursaphelenchus xylophilus*. *FEBS Lett.* **572**, 201-205 (2004).

22. Kikuchi, T., Shibuya, H. & Jones, J. T. Molecular and biochemical characterization of an endo-beta-1,3-glucanase from the pinewood nematode *Bursaphelenchus xylophilus* acquired by horizontal gene transfer from bacteria. *Biochemical. J.* **389**, 117-125 (2005).

23. Ding, X., Shields, J., Allen, R. & Hussey, R. S. A secretory cellulose-binding protein cDNA cloned from the root-knot nematode (*Meloidogyne incognita*). *Mol. Plant-Microbe Interact.* **11**, 952-959 (1998).

24. Gao, B., Allen, R., Davis, E. L., Baum, T. J. & Hussey, R. S. Molecular characterisation and developmental expression of a cellulose-binding protein gene in the soybean cyst nematode *Heterodera glycines*. *Int. J. Parasitol.* **34**, 1377-1383 (2004).

25. Adam, M., Phillips, M. S., Jones, J. T. & Blok, V. C. Characterisation of the cellulose-binding protein Mj-cbp-1 of the root knot nematode, *Meloidogyne javanica*. *Physiol. Mol. Plant Pathol.* **72**, 21-28 (2008).

26. Hewezi, T. *et al*. Cellulose binding protein from the parasitic nematode *Heterodera schachtii* interacts with *Arabidopsis* pectin methylesterase: cooperative cell wall modification during parasitism. *Plant Cell* **20**, 3080-3093 (2008).

27. Kikuchi, T., Shibuya, H., Aikawa, T. & Jones, J. T. Cloning and characterization of pectate lyases expressed in the esophageal gland of the pine wood nematode *Bursaphelenchus xylophilus*. *Mol. Plant-Microbe Interact.* **19**, 280-287 (2006).

28. Popeijus, M. *et al*. Analysis of genes expressed in second stage juveniles of the potato cyst nematodes *Globodera rostochiensis* and *G. pallida* using the expressed sequence tag approach. *Nematology* **2**, 567-574 (2000).

29. Doyle, E. A. & Lambert, K. N. *Meloidogyne javanica* chorismate mutase 1 alters plant cell development. *Mol. Plant-Microbe Interact.* **16**, 123-131 (2003).

30. Huang, G. *et al*. Developmental expression and molecular analysis of two *Meloidogyne incognita* pectate lyase genes. *Int. J. Parasitol.* **35**, 685-692 (2005).

31. de Boer, J. M. *et al*. The use of DNA microarrays for the developmental expression analysis of cDNAs from the oesophageal gland cell region of *Heterodera glycines*. *Mol. Plant Pathol.* **3**, 261-270 (2002).

32. Vanholme, B. *et al*. Molecular characterization and functional importance of pectate lyase secreted by the cyst nematode *Heterodera schachtii*. *Mol. Plant Pathol.* **8**, 267-278 (2007).

33. Vanholme, B., Haegeman, A., Jacob, J., Cannoot, B. & Gheysen, G. Arabinogalactan endo-1,4-beta-galactosidase: a putative plant cell wall-degrading enzyme of plant-parasitic nematodes. *Nematology* **11**, 739-747 (2009).

34. Jaubert, S. *et al*. Direct identification of stylet secreted proteins from root-knot nematodes by a proteomic approach. *Mol. Biochem. Parasitol.* **121**, 205-211 (2002).

35. Mitreva-Dautova, M. *et al*. A symbiont-independent endo-1,4-beta-xylanase from the plant-parasitic nematode *Meloidogyne incognita*. *Mol. Plant-Microbe Interact.* **19**, 521-529 (2006).

36. Haegeman, A., Vanholme, B. & Gheysen, G. Characterization of a putative endoxylanase in the migratory plant-parasitic nematode *Radopholus similis*. *Mol. Plant Pathol.* **10**, 389-401 (2009).

37. Kikuchi, T. *et al*. Identification of putative expansin-like genes from the pine wood nematode, *Bursaphelenchus xylophilus*, and evolution of the expansin gene family within the Nematoda. *Nematology* **11**, 355-364 (2009).

38. Kudla, U. *et al*. Origin, distribution and 3D-modeling of Gr-EXPB1, an expansin from the potato cyst nematode *Globodera rostochiensis*. *FEBS Lett.* **579**, 2451-2457 (2005).

39. Qin, L. *et al*. Plant degradation: a nematode expansin acting on plants. *Nature* **427**, 30, (2004).

40. Prior, A. *et al*. A surface-associated retinol- and fatty acid-binding protein (Gp-FAR-1) from the potato cyst nematode *Globodera pallida*: lipid binding activities, structural analysis and expression pattern. *Biochemical. J.* **356**, 387-394 (2001).

41. Bellafiore, S. *et al*. Direct identification of the *Meloidogyne incognita* secretome reveals proteins with host cell reprogramming potential. *PLoS Pathog.* **4**, e1000192, doi: 10.1371/journal.ppat.1000192 (2008).

42. Robertson, L. *et al*. Cloning, expression and functional characterisation of a peroxiredoxin from the potato cyst nematode *Globodera rostochiensis*. *Mol. Biochem. Parasitol.* **111**, 41-49 (2000).

43. Dubreuil, G. *et al*. Peroxiredoxins from the plant parasitic root-knot nematode, *Meloidogyne incognita*, are required for successful development within the host. *Int. J. Parasitol.* **41**, 385-396 (2011).

44. Jones, J. T., Reavy, B., Smant, G. & Prior, A. E. Glutathione peroxidases of the potato cyst nematode *Globodera rostochiensis*. *Gene* **324**, 47-54 (2004).

45. Dubreuil, G., Magliano, M., Deleury, E., Abad, P. & Rosso, M. N. Transcriptome analysis of root-knot nematode functions induced in the early stages of parasitism. *New Phytol.* **176**, 426-436 (2007).

46. Haegeman, A. *et al*. Expressed sequence tags of the peanut pod nematode *Ditylenchus africanus*: The first transcriptome analysis of an Anguinid nematode. *Mol. Biochem. Parasitol.* **167**, 32-40 (2009).

47. Bakhetia, M., Urwin, P. E. & Atkinson, H. J. qPCR analysis and RNAi define pharyngeal gland cell-expressed genes of *Heterodera glycines* required for initial interactions with the host. *Mol. Plant-Microbe Interact.* **20**, 306-312 (2007).

48. Gao, B. *et al*. The parasitome of the phytonematode *Heterodera glycines*. *Mol. Plant-Microbe Interact.* **16**, 720-726 (2003).

49. Bekal, S., Niblack, T. L. & Lambert, K. N. A chorismate mutase from the soybean cyst nematode *Heterodera glycines* shows polymorphisms that correlate with virulence. *Mol. Plant-Microbe Interact.* **16**, 439-446 (2003).

50. Jones, J. T. *et al*. Characterization of a chorismate mutase from the potato cyst nematode *Globodera pallida*. *Mol. Plant Pathol.* **4**, 43-50 (2003).

51. Huang, G. *et al*. Two chorismate mutase genes from the root-knot nematode *Meloidogyne incognita*. *Mol. Plant Pathol.* **6**, 23-30 (2005).

52. Vanholme, B. *et al*. Structural and functional investigation of a secreted chorismate mutase from the plant-parasitic nematode *Heterodera schachtii* in the context of related enzymes from diverse origins. *Mol. Plant Pathol.* **10**, 189-200 (2009).

53. Long, H., Wang, X. & Xu, J. Molecular cloning and life-stage expression pattern of a new chorismate mutase gene from the root-knot nematode *Meloidogyne arenaria*. *Plant Pathol.* **55**, 559-563 (2006).

54. Long, H., Wang, X., Xu, J. H. & Hu, Y. J. Isolation and characterization of another cDNA encoding a chorismate mutase from the phytoparasitic nematode *Meloidogyne arenaria*. *Exp. Parasitol.* **113**, 106-111 (2006).

55. Lu, S., Tian, D., Borchardt-Wier, H. B. & Wang, X. Alternative splicing: a novel mechanism of regulation identified in the chorismate mutase gene of the potato cyst nematode *Globodera rostochiensis*. *Mol. Biochem. Parasitol.* **162**, 1-15 (2008).

56. Blanchard, A., Esquibet, M., Fouville, D. & Grenier, E. Ranbpm homologue genes characterised in the cyst nematodes *Globodera pallida* and *Globodera mexicana*. *Physiol. Mol. Plant Pathol.* **67**, 15-22 (2005).

57. Sacco, M. A. *et al*. The cyst nematode SPRYSEC protein RBP-1 elicits Gpa2- and RanGAP2-dependent plant cell death. *PLoS Pathog.* **5**, e1000564, doi: 10.1371/journal.ppat.1000564 (2009).

58. Lu, S. W. *et al*. Structural and functional diversity of CLAVATA3/ESR (CLE)-like genes from the potato cyst nematode *Globodera rostochiensis*. *Mol. Plant-Microbe Interact.* **22**, 1128-1142 (2009).

59. Wang, J. *et al*. Identification of potential host plant mimics of CLAVATA3/ESR (CLE)-like peptides from the plant-parasitic nematode *Heterodera schachtii*. *Mol. Plant Pathol.* **12**, 177-186 (2011).

60. Wang, X. *et al*. A parasitism gene from a plant-parasitic nematode with function similar to CLAVATA3/ESR (CLE) of *Arabidopsis thaliana*. *Mol. Plant Pathol.* **6**, 187-191 (2005).

61. Huang, G., Allen, R., Davis, E. L., Baum, T. J. & Hussey, R. S. Engineering broad root-knot resistance in transgenic plants by RNAi silencing of a conserved and essential root-knot nematode parasitism gene. *Proc. Natl. Acad. Sci. USA* **103**, 14302-14306 (2006).

62. Huang, G. *et al*. A root-knot nematode secretory peptide functions as a ligand for a plant transcription factor. *Mol. Plant-Microbe Interact.* **19**, 463-470 (2006).

63. Lee, C. *et al*. The novel cyst nematode effector protein 19C07 interacts with the *Arabidopsis* auxin influx transporter LAX3 to control feeding site development. *Plant Physiol.* **155**, 866-880 (2011).

64. Opperman, C. H. *et al*. Sequence and genetic map of *Meloidogyne hapla*: a compact nematode genome for plant parasitism. *Proc. Natl. Acad. Sci. USA* **105**, 14802-14807 (2008).

65. Patel, N. *et al*. A nematode effector protein similar to annexins in host plants. *J. Exp. Bot.* **61**, 235-248 (2010).

66. Jaubert, S., Laffaire, J. B., Abad, P. & Rosso, M. N. A polygalacturonase of animal origin isolated from the root-knot nematode *Meloidogyne incognita*. *FEBS Lett.* **522**, 109-112 (2002).

67. Jaubert, S. *et al*. *In planta* secretion of a calreticulin by migratory and sedentary stages of root-knot nematode. *Mol. Plant-Microbe Interact.* **18**, 1277-1284 (2005).

68. Jaubert, S. *et al*. Comparative analysis of two 14-3-3 homologues and their expression pattern in the root-knot nematode *Meloidogyne incognita*. *Int. J. Parasitol.* **34**, 873-880 (2004).

69. Hewezi, T. *et al*. *Arabidopsis* spermidine synthase is targeted by an effector protein of the cyst nematode *Heterodera schachtii*. *Plant Physiol.* **152**, 968-984 (2010).

70. Tytgat, T. *et al*. A new class of ubiquitin extension proteins secreted by the dorsal pharyngeal gland in plant parasitic cyst nematodes. *Mol. Plant-Microbe Interact.* **17**, 846-852 (2004).

71. Karim, N., Jones, J. T., Okada, H. & Kikuchi, T. Analysis of expressed sequence tags and identification of genes encoding cell-wall-degrading enzymes from the fungivorous nematode *Aphelenchus avenae*. *BMC Genomics* **10**, 525, doi: 10.1186/1471-2164-10-525 (2009).

72. Kikuchi, T. *et al*. Expressed sequence tag (EST) analysis of the pine wood nematode *Bursaphelenchus xylophilus* and *B. mucronatus*. *Mol. Biochem. Parasitol.* **155**, 9-17 (2007).

73. Gao, B. *et al*. Characterisation and developmental expression of a chitinase gene in *Heterodera glycines*. *Int. J. Parasitol.* **32**, 1293-300 (2002).

74. Huang, G. *et al*. A profile of putative parasitism genes expressed in the esophageal gland cells of the root-knot nematode *Meloidogyne incognita*. *Mol. Plant-Microbe Interact.* **16**, 376-381 (2003).

75. Jacob, J., Vanholme, B., Haegeman, A. & Gheysen, G. Four transthyretin-like genes of the migratory plant-parasitic nematode *Radopholus similis*: Members of an extensive nematode-specific family. *Gene* **402**, 9-19 (2007).

76. Semblat, J. P., Rosso, M. N., Hussey, R. S., Abad, P. & Castagnone-Sereno, P. Molecular cloning of a cDNA encoding an amphid-secreted putative avirulence protein from the root-knot nematode *Meloidogyne incognita*. *Mol. Plant-Microbe Interact.* **14**, 72-79 (2001).

77. Abad, P. *et al*. Genome sequence of the metazoan plant-parasitic nematode *Meloidogyne incognita*. *Nat. Biotechnol.* **26**, 909 (2008).

78. de Boer, J. M. *et al.* Cloning of a putative pectate lyase gene expressed in the subventral esophageal glands of *Heterodera glycines*. *J. Nematol.* **34**, 9-11 (2002).

79. Vanholme, B. *et al*. Detection of putative secreted proteins in the plant-parasitic nematode *Heterodera schachtii*. *Parasitol. Res.* **98**, 414-424 (2006).

80. Gao, B. *et al*. Molecular characterisation and expression of two venom allergen-like protein genes in *Heterodera glycines*. *Int. J. Parasitol.* **31**, 1617-1625 (2001).

81. Ding, X., Shields, J., Allen, R. & Hussey, R. S. Molecular cloning and characterisation of a venom allergen AG5-like cDNA from *Meloidogyne incognita*. *Int. J. Parasitol.* **30**, 77-81 (2000).

82. Wang, X., Li, H., Hu, Y., Fu, P. & Xu, J. Molecular cloning and analysis of a new venom allergen-like protein gene from the root-knot nematode *Meloidogyne incognita*. *Exp. Parasitol.* **117**, 133-140 (2007).

83. Jones, J. T., Smant, G. & Blok, V. C. SXPRAL-2 proteins of the potato cyst nematode *Globodera rostochiensis* secreted proteins of the hypodermis and amphids. *Nematology* **2**, 887-893 (2000).

84. Tytgat, T. *et al*. An SXP/RAL-2 protein produced by the subventral pharyngeal glands in the plant parasitic root-knot nematode *Meloidogyne incognita*. *Parasitol. Res.* **95**, 50-54 (2005).

85. Wang, X. *et al*. Signal peptide-selection of cDNA cloned directly from the esophageal gland cells of the soybean cyst nematode *Heterodera glycines*. *Mol. Plant-Microbe Interact.* **14**, 536-544 (2001).

86. Liu, J., Koltai, H., Chejanovsky, N. & Spiegel, Y. Isolation of a novel collagen gene (*Mj-col-5*) in *Meloidogyne javanica* and analysis of its expression pattern. *J. Parasitol.* **87**, 801-807 (2001).

87. Castagnone-Sereno, P., Leroy, F. & Abad, P. cDNA cloning and expression analysis of a calponin gene from the plant-parasitic nematode *Meloidogyne incognita*. *Mol. Biochem. Parasitol.* **112**, 149-152 (2001).

88. Hu, L. *et al*. Molecular and biochemical characterization of the β-1,4-endoglucanase gene *Mj-eng-3* in the root-knot nematode *Meloidogyne javanica*. *Exp. Parasitol.* **135**, 15-23 (2013).

89. Xie, J. *et al*. A novel *Meloidogyne incognita* effector misp12 suppresses plant defense response at latter stages of nematode parasitism. *Front. Plant Sci.* **7**, 964, doi: 10.3389/fpls.2016.00964 (2016).

90. Huang, G. *et al*. Use of solid-phase subtractive hybridization for the identification of parasitism gene candidates from the root-knot nematode *Meloidogyne incognita*. *Mol. Plant Pathol.* **5**, 217-222 (2004).

91. Gleason, C. A., Liu, Q. L. & Williamson, V. M. Silencing a candidate nematode effector gene corresponding to the tomato resistance gene *Mi-1* leads to acquisition of virulence. *Mol. Plant-Microbe Interact.* **21**, 576-585 (2008).

92. Ray, C., Abbott, A. G. & Hussey, R. S. Trans-splicing of a *Meloidogyne incognita* mRNA encoding a putative esophageal gland protein. *Mol. Biochem. Parasitol.* **68**, 93-101 (1994).

93. Wang, T., Deom, C. M. & Hussey, R. S. Identification of a *Meloidogyne incognita* cuticle collagen gene and characterization of the developmental expression of three collagen genes in parasitic stages. *Mol. Biochem. Parasitol.* **93**, 131-134 (1998).

94. Noon, J. B. *et al*. Eighteen new candidate effectors of the phytonematode *Heterodera glycines* produced specifically in the secretory esophageal gland cells during parasitism. *Phytopathology* **105**, 1362-1372 (2015).

95. Zhuo, K. *et al*. A novel *Meloidogyne enterolobii* effector MeTCTP promotes parasitism by suppressing programmed cell death in host plants. *Mol. Plant Pathol.* **18**, 45, doi: 10.1111/mpp.12374 (2016).

96. Lin, B. *et al*. A novel nematode effector suppresses plant immunity by activating host reactive oxygen species-scavenging system. *New Phytol.* **209**, 1159-1173 (2016).

97. Wubben, M. J., Gavilano, L., Baum, T. J. & Davis, E. L. Sequence and spatiotemporal expression analysis of CLE-motif containing genes from the reniform nematode (*Rotylenchulus reniformis* Linford & Oliveira). *J. Nematol.* **47**, 159-165 (2015).

98. Li, Y. *et al*. A nematode calreticulin, Rs-CRT, is a key effector in reproduction and pathogenicity of *Radopholus similis*. *PLoS ONE* **10**, e129351, doi: 10.1371/journal.pone.0129351 (2015).

99. Chen, C. *et al*. An ANNEXIN-like protein from the cereal cyst nematode *Heterodera avenae* suppresses plant defense. *PLoS ONE* **10**, e122256, doi: 10.1371/journal.pone.0122256 (2015).

100. Hewezi, T. *et al*. The cyst nematode effector protein 10A07 targets and recruits host posttranslational machinery to mediate its nuclear trafficking and to promote parasitism in *Arabidopsis*. *Plant Cell* **27**, 891-907 (2015).

101. Eves-van den Akker, S., Lilley, C. J., Jones, J. T. & Urwin, P. E. Identification and characterisation of a hyper-variable apoplastic effector gene family of the potato cyst nematodes. *PLoS Pathog.* **10**, e1004391, doi: 10.1371/journal.ppat.1004391 (2014).

102. Dinh, P. T., Brown, C. R. & Elling, A. A. RNA interference of effector gene *Mc16D10L* confers resistance against *Meloidogyne chitwoodi* in *Arabidopsis* and potato. *Phytopathology* **104**, 1098-1106 (2014).

103. Chronis, D. *et al*. A ubiquitin carboxyl extension protein secreted from a plant-parasitic nematode *Globodera rostochiensis* is cleaved *in planta* to promote plant parasitism. *Plant J.* **74**, 185-196 (2013).

104. Rehman, S. *et al*. A secreted SPRY domain-containing protein (SPRYSEC) from the plant-parasitic nematode *Globodera rostochiensis* interacts with a CC-NB-LRR protein from a susceptible tomato. *Mol. Plant-Microbe Interact.* **22**, 330-340 (2009).

105. Lin, B. *et al*. A novel effector protein, MJ-NULG1a, targeted to giant cell nuclei plays a role in *Meloidogyne javanica* parasitism. *Mol. Plant-Microbe Interact.* **26**, 55-66 (2013).

106. Lozano-Torres, J. L. *et al*. Dual disease resistance mediated by the immune receptor Cf-2 in tomato requires a common virulence target of a fungus and a nematode. *Proc. Natl. Acad. Sci. USA* **109**, 10119-10124 (2012).

107. Eves-van den Akker, S., Lilley, C. J., Yusup, H. B., Jones, J. T. & Urwin, P. E. Functional C-TERMINALLY ENCODED PEPTIDE (CEP) plant hormone domains evolved *de novo* in the plant parasite *Rotylenchulus reniformis*. *Mol. Plant Pathol.* **17**, 1265-1275 (2016).
